# Cooperation of Adhesin Alleles in *Salmonella*-Host Tropism

**DOI:** 10.1128/mSphere.00066-17

**Published:** 2017-03-08

**Authors:** Leon De Masi, Min Yue, Changmin Hu, Alexey V. Rakov, Shelley C. Rankin, Dieter M. Schifferli

**Affiliations:** Department of Pathobiology, University of Pennsylvania, School of Veterinary Medicine, Philadelphia, Pennsylvania, USA; University of Oslo

**Keywords:** *Salmonella* Newport, adhesins, allelic variation, fimbriae, host tropism

## Abstract

Allelic combinations and host specificities for three fimbrial adhesins, FimH, BcfD, and StfH, were compared for 262 strains of *Salmonella enterica* serovar Newport, a frequent human and livestock pathogen. Like FimH, BcfD had two major alleles (designated A and B), whereas StfH had two allelic groups, each with two alleles (subgroup A1 and A2 and subgroup B1 and B2). The most prevalent combinations of FimH/BcfD/StfH alleles in *S*. Newport were A/A/A1 and B/B/B1. The former set was most frequently found in bovine and porcine strains, whereas the latter combination was most frequently found in environmental and human isolates. Bacteria genetically engineered to express Fim, Bcf, or Stf fimbriae on their surface were tested with the different alleles for binding to human, porcine, and bovine intestinal epithelial cells. The major allelic combinations with bovine and porcine strains (A/A/A1) or with human isolates (B/B/B1) provided at least two alleles capable of binding significantly better than the other alleles to an intestinal epithelial cell line from the respective host(s). However, each combination of alleles kept at least one allele mediating binding to an intestinal epithelial cell from another host. These findings indicated that allelic variation in multiple adhesins of *S*. Newport contributes to bacterial adaptation to certain preferential hosts without losing the capacity to maintain a broad host range.

**IMPORTANCE** *Salmonella enterica* remains a leading foodborne bacterial pathogen in the United States; infected livestock serve often as the source of contaminated food products. A study estimated that over a billion *Salmonella* gastroenteritis cases and up to 33 million typhoid cases occur annually worldwide, with 3.5 million deaths. Although many *Salmonella* strains with a broad host range present preferential associations with certain host species, it is not clear what determines the various levels of host adaptation. Here, causal properties of host associations were determined with allelic variants of three colonization factors of *S. enterica* serovar Newport, a most frequent zoonotic serovar. This is the first study that related not only individual but also a small group of host-associated gene variants with functional properties that cooperate to determine the level of host-adapted virulence. The detected associations should help to identify sources of *Salmonella* infections in both humans and animals.

## Abstract (precis)

*Salmonella enterica* remains a leading foodborne bacterial pathogen in the United States; infected livestock serve often as the source of contaminated food products. A study estimated that over a billion *Salmonella* gastroenteritis cases and up to 33 million typhoid cases occur annually worldwide, with 3.5 million deaths. Although many *Salmonella* strains with a broad host range present preferential associations with certain host species, it is not clear what determines the various levels of host adaptation. Here, causal properties of host associations were determined with allelic variants of three colonization factors of *S. enterica* serovar Newport, a most frequent zoonotic serovar. This is the first study that related not only individual but also a small group of host-associated gene variants with functional properties that cooperate to determine the level of host-adapted virulence. The detected associations should help to identify sources of *Salmonella* infections in both humans and animals.

## INTRODUCTION

*Salmonella enterica* subsp. *enterica* (*S. enterica*) bacteria are persistent infectious agents, colonizing the intestinal tract of humans and animals worldwide (1). Each year, *Salmonella enterica* is responsible for 12 to 33 million human typhoid cases (2) and 1.3 billion cases of gastroenteritis worldwide (3), with approximately 3.5 million deaths. It is frequently the leading cause of foodborne infections in the United States annually (4) and a persistent agricultural problem, as *Salmonella* strains infect livestock (5); this in turn exposes humans to contaminated food sources (6). *S. enterica* subsp. *enterica* uses a variety of virulence factors during the course of infection (7), including a series of effectors secreted by two different type III secretion systems (8), flagella (9), and a series of nonfimbrial and fimbrial adhesins (10). Many characterized adhesins are expressed on the structures of chaperone-usher fimbriae (11).

A recent study on available *Salmonella* genomes detected 35 different fimbrial gene clusters (11), and although some have been shown to participate in murine intestinal colonization using mutants (12, 13), little is known about their potential function in colonizing the intestinal epithelia of various hosts. The presence of genes for at least two fimbrial subunits in each cluster suggests that they would form heteropolymeric structures with tip adhesins, according to the current model for the type 1 and P fimbriae of *Escherichia coli* (14). Unlike the *Salmonella* type 1 fimbriae (Fim), which can be detected by bacterial agglutination with corresponding antibodies or visualized on bacterial surfaces by electron microscopy, most fimbrial chaperone usher gene clusters of *Salmonella* are not expressed under standard laboratory conditions. However, some gene clusters can be induced to express fimbriae, typically as recombinant proteins (15, 16).

Fimbriae mediate bacterial binding to the surface of eukaryotic cells that carry cognate receptors (17, 18). In an earlier study, we showed that the type 1 fimbriae of *S. enterica* serovar Typhimurium mediate bacterial binding in a host-specific manner thanks to adhesin alleles that are adapted to distinct host receptors (19). In addition, we observed that predicted protein sequences of S. enterica serovar Newport FimH could distinguish two major groups of FimH alleles, depending on whether they were of bovine origin (group A) or not (group B) (20). That study also revealed that bovine isolates with the typical FimH allele mostly carried genes for one of two alleles for the predicted Bcf and Stf adhesins.

The *fim* and *bcf* gene clusters are conserved in all *S. enterica* bacteria, and while the *stf* gene cluster is present in many serovars of *S. enterica*, including Typhimurium and Newport, it is absent in others, such as Typhi, Montevideo, and Schwarzengrund (11, 21, 22). In contrast to the *Salmonella* type 1 fimbria Fim, the *bcf* and *stf* gene clusters encode cryptic fimbriae that have not yet been visualized on bacteria. Data from a transposon mutant studied in orally infected calves identified the involvement of Bcf as a bovine colonization factor (23). The predicted major fimbrial subunit BcfA could be expressed in bovine ligated ileal loops (24), and a *bcf* mutant served to illustrate the contribution of Bcf, together with other cryptic fimbriae, to intestinal persistence in mice (25). Bcf expression in mice was inferred when mice experimentally infected with S. Typhimurium produced antibodies against BcfA, as well as against the FimA and StfA fimbrial subunits, together with subunits of other fimbriae (26). A *bcf* mutant produced more biofilm on human epithelial cells or chicken intestinal tissue (27), suggesting that Bcf interferes with the expression or function of other fimbriae. The orthologous *E. coli* gene clusters for *bcf* and *stf* are the *ycb* and *yfc* fimbrial genes, respectively (28, 29). Ycb fimbriae were detectable after the insertion of a constitutive promoter and contributed to biofilms in the absence of Fim (28). Although Yfc fimbriae of *E. coli* could not be detected after forced expression of *yfc*, the bacteria bound 10 times better to T24 bladder cells (28). The presence of *yfc* genes was associated with uropathogenic *E. coli* (30), and their presence contributed to murine bladder colonization (31). These results suggested that the corresponding *Salmonella bcf* and *stf* genes should be capable of producing fimbrial structures that might have some adhesive phenotype.

Here, we showed that Bcf and Stf fimbriae can be expressed as bacterial surface organelles. We demonstrated that a large majority of bovine and porcine isolates of *S*. Newport carry mainly one particular set of *fimH*, *bcfD*, and *stfH* adhesin alleles, while *S*. Newport isolates from humans, horses, and chickens possess more-diversified distributions of alleles. By investigating the properties of binding of different Bcf and Stf adhesin alleles to intestinal epithelial cells, we demonstrated that allelic variants of both fimbriae, associated with strains isolated from specific host species, mediate preferential bacterial adhesion to the same host species. Some binding was also selective for epithelial cells from specific intestinal segments. Together with FimH, allelic combinations among the three adhesins showed either additive binding specificity for one or a few host species or some complementary binding affinities for a broader spectrum of hosts. These data suggested that distinct sets of fimbrial adhesin alleles from broad-host-range *Salmonella* serovars such as *S*. Newport can coevolve to contribute to preferential host-species adaptation.

## RESULTS

### Major BcfD, StfH, and FimH alleles in *S*. Newport.

**FIG 1:**
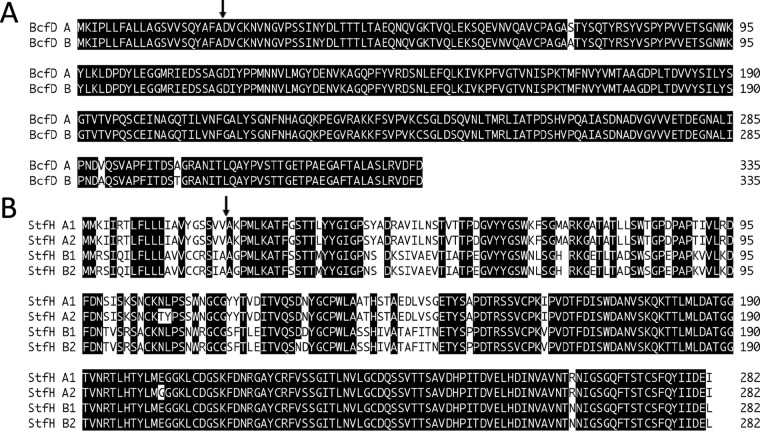
Protein sequence alignments for the BcfD (A) and StfH (B) allelic groups from *S*. Newport. Alignments were made using ClustalW (MegAlign; DNAStar, Madison, WI). Identical amino acids are shadowed in black with white text, while different amino acids appear as black text with no shadowing. Allelic sequence labels are indicated at left and amino acid positions at right. Arrows indicate predicted signal sequence cleavage sites.

On the basis of a previous targeted sequencing study that offered preliminary data on allelic variations for eight known or predicted fimbrial adhesin genes of 46 *S*. Newport isolates (20), we observed that most adhesins could be separated into two major allelic groups (designated A and B). However, since half of the strains were of bovine origin, these results needed to be reevaluated with *S*. Newport strains isolated from different sources in larger numbers. Here, we reanalyzed the sequences of these adhesins by investigating available data for 281 *S*. Newport isolates of bovine, equine, porcine, avian, human, and environmental origin. Our earlier findings on the presence of two major allelic groups (A and B) for each of three adhesins were confirmed with 262 strains (Table S1). Adhesin sequence information was missing or incomplete for 11 raw genomic data files. Moreover, we excluded from this study the adhesin sequences of eight strains with slight variations for the group A and/or B alleles, since their numbers were too low for significant evaluation. Protein sequence alignments for groups A and B of the 262 investigated strains revealed two- or three-residue differences at positions 32 and 89 for FimH (19) or positions 72, 289, and 300 for BcfD (Fig. 1A). The StfH alleles revealed multiple differences between groups A and B, particularly in the N-terminal putative lectin-binding domain (Fig. 1B). An additional few sequence differences within groups A and B of StfH further divided them into 2 subgroups each (subgroup A1 and A2 and subgroup B1 and B2).

10.1128/mSphere.00066-17.4 TABLE S1  *S*. Newport strains and corresponding metadata. Download TABLE S1, PDF file, 0.2 MB. Copyright © 2017 De Masi et al. 2017 De Masi et al. This content is distributed under the terms of the Creative Commons Attribution 4.0 International license.

### Bacterial origin correlates with distinct groups of adhesin alleles.

**FIG 2:**
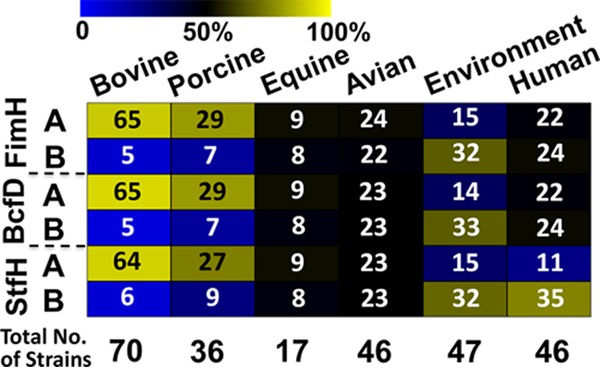
Association of *S*. Newport strain sources with adhesin alleles. The percentages of *S*. Newport strains for which the source of isolation was associated with a specific adhesin allele (group A or B) are displayed as colors, based on a gradient from blue (0% association) to black (50% association) and yellow (100% association). The exact numbers of strains are indicated in each cell, and total numbers for each source of isolation are shown at the bottom of the heat map.

Our previous study on *S*. Newport adhesin alleles had revealed that one FimH allelic group associated with strains from bovine sources, suggesting some host adaptation (20). Despite a convergent trend, a corresponding association for the other two adhesins was not significant, likely due to the diverse origins of the nonbovine strains available for the analysis. Here, we reinvestigated the FimH, BcfD, and StfH A and B allelic groups of the 262 strains of *S*. Newport by host origin. There were significant differences between the isolates from certain hosts and from the environment in the distributions of group A and group B adhesins (Fig. 2; see also Fig. S1 and S2 and Table S2A and B and C in the supplemental material). The members of group A of all three adhesins were mainly associated with bovine and porcine isolates, compared to other hosts or the environment. Over 95% of the StfH group A strains consisted of subgroup A1 alleles, while just 6 isolates had StfH A2 alleles (Fig. S1 and S2) (Table S2C). Equine and avian strains were split evenly between groups A and B, while environmental strains were predominantly in group B. Human strains were evenly distributed in the two groups for FimH and BcfD but were more numerous in StfH group B, as with the environmental strains. Most group B strains belonged to subgroup B1, particularly among the environmental strains (Fig. S1 and S2) (Table S2C).

10.1128/mSphere.00066-17.1 FIG S1  Association of *S*. Newport strain sources with adhesin alleles. The percentages of *S*. Newport strains for which the source of isolation was associated with a specific adhesin allele (group A or B for FimH and BcfD; subgroup A1, A2, B1, or B2 for StfH) are displayed as colors, based on a gradient from blue (0% association) to black (50% association) and yellow (100% association). The exact numbers of strains are indicated in each cell, and total numbers for each source of isolation are shown at the bottom of the heat map. Download FIG S1, PDF file, 0.3 MB. Copyright © 2017 De Masi et al. 2017 De Masi et al. This content is distributed under the terms of the Creative Commons Attribution 4.0 International license.

10.1128/mSphere.00066-17.2 FIG S2  Numbers of *S*. Newport isolates for each adhesin allele and for each strain source. (Top left panel) Strains with allele A or B of FimH. (Top right panel) Strains with allele A or B of BcfD. (Bottom left panel) Strains with allele A or B of StfH. (Bottom right panel) Strains with allele A1, A2, B1, or B2 of StfH. Significant different distributions of allelic groups between two sources of strains were determined by the Fisher’s exact test (see Tables S3 to S5); levels of statistical significance are shown as stars (*, *P* < 0.05; **, P < 00.1; ***, *P* < 0.001) below brackets, with an open end indicating a given source and a closed end indicating a compared source. Download FIG S2, PDF file, 0.3 MB. Copyright © 2017 De Masi et al. 2017 De Masi et al. This content is distributed under the terms of the Creative Commons Attribution 4.0 International license.

10.1128/mSphere.00066-17.5 TABLE S2  Statistical 2 × 2 contingency table data (*P* values [Fisher’s exact test]), with strain numbers separated by sources and FimH (A), BcfD (B), or StfH (C) alleles. Download TABLE S2, PDF file, 0.02 MB. Copyright © 2017 De Masi et al. 2017 De Masi et al. This content is distributed under the terms of the Creative Commons Attribution 4.0 International license.

**FIG 3:**
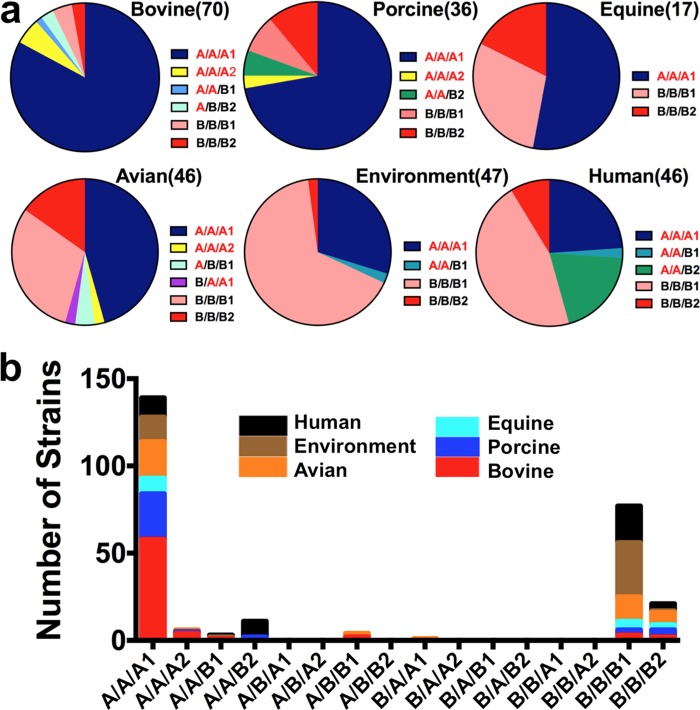
Combinations of adhesin alleles for each isolation source. (a) Relative frequencies of *S*. Newport strains, with each detected combination of adhesin alleles grouped by isolation source. The detected allele combinations of FimH/BcfD/StfH for each source are listed on the right of the corresponding pie charts. The following colors were used for the different combinations: dark blue (A/A/A1), medium blue (A/A/B1), light blue (A/B/B1), green (A/A/B2), pink (B/B/B1), red (B/B/B2), and purple (B/A/A1). Six strains with an StfH group A2 allele (four bovine strains, one porcine strain, and one avian strain) were excluded from the charts. (b) Numbers of each combination of alleles are shown with stacked columns, with each color representing the corresponding number for one source of isolates (human, black; environment, brown; avian, orange; equine, light blue; porcine, blue; bovine, red).

Preferential host or niche association with allelic combinations for the three adhesins was even more specific (Fig. 3), in agreement with our earlier observation of allelic group overlaps that suggested the presence of linkage disequilibrium (20). Most strains had either the FimH/BcfD/StfH A/A/A1 or the B/B/B1 combination. The former combination was associated with bovine and porcine sources, whereas the latter one was mainly found with environmental or human strains. The B/B/B2 combination was the third-most-frequent one, whereas other combinations were rarer, with the exception of A/A/B2 strains that were specifically associated with humans and represented 20% of their isolates. In addition, the bovine/porcine strains with the frequent FimH/BcfD/StfH A/A/A1 alleles were isolated over 16 years, and the human/environmental strains with the other frequent alleles (the B/B/B1 and B/B/B2 alleles) were isolated over 16 and 14 years, respectively. Strains with each of these alleles were also isolated concurrently at distinct locations (Europe and different states within the United States), strongly suggesting that the porcine and bovine strains were not epidemic clones temporally or geographically separated from human or environmental strains.

Taking the data together, different distributions of *S*. Newport alleles of the three adhesins alone or in combination were associated with specific hosts or the environment. Strains with the three adhesins from group A were typically bovine or porcine isolates, while strains with three adhesins from group B were more likely isolated from the environment. Most human strains had an StfH group B allele, like most environmental strains, although their FimH and BcfD alleles included comparable numbers of group A and B alleles.

### Production of Bcf and Stf fimbriae.

**FIG 4:**
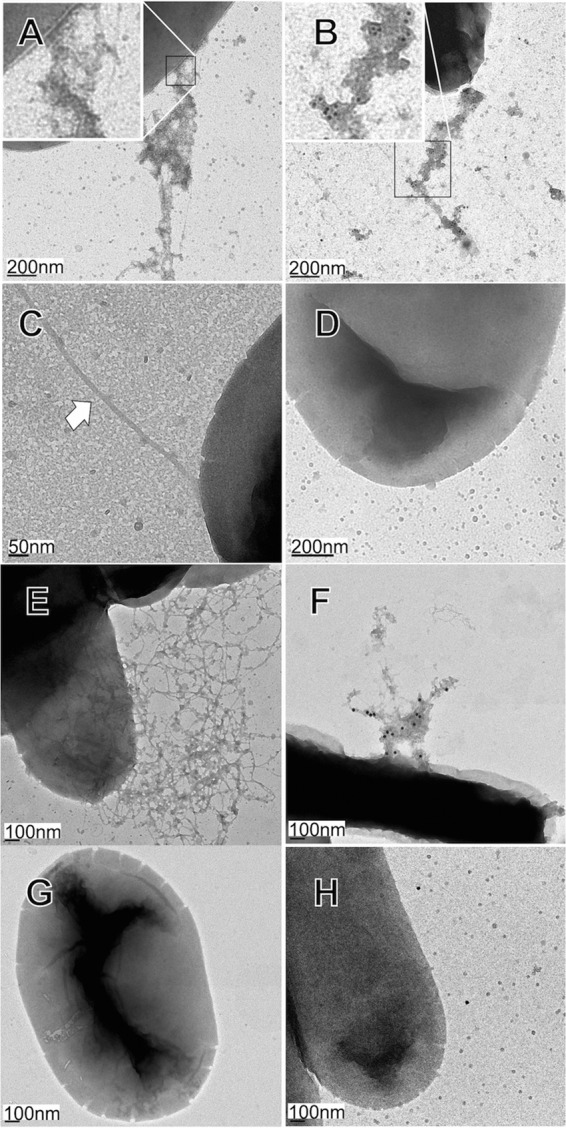
Transmission electron microscopy of Bcf- and Stf-fimbriated bacteria. (A) *E. coli* AAEC189 pLDBAD-Stf, after Stf induction. The inset is a close-up image of fimbriae and fimbrial aggregates at the bacterial cell surface. (B) Immunogold-labeled Stf fimbriae and fimbrial aggregates on *E. coli* SE5000 containing pLDBAD-Stf using adsorbed rabbit anti-Stf antisera, as described in Materials and Methods, followed by anti-rabbit antibodies conjugated to 10-nm-diameter gold particles. The inset is a close-up image showing numerous gold particles attached to fimbrial aggregates. (C) *E. coli* AAEC189 pBAD33 (empty vector control). The white arrow denotes a flagellum (flagella were absent from SE5000). (D) Immunogold labeling of *E. coli* SE5000 pBAD33, as described for panel B. (E) *E. coli* AEEC189 pLDHSG-Bcf-S after Bcf induction. (F) Immunogold-labeled strain AJB4Δ*bcfC* pLDHSG-Bcf-L processed with adsorbed rabbit anti-Bcf antisera followed by anti-rabbit antibodies conjugated to 20-nm-diameter gold particles, showing entangled fimbriae with numerous gold particles attached. (G) *E. coli* SE5000 pHSG576 (empty vector control). (H) Immunogold labeling of strain AJB4Δ*bcfC* pHSG576, as described for panel F.

The Bcf and Stf gene clusters from *S*. Newport strain SL254 were cloned into the pHSG-576 and pBAD33 expression vectors, respectively. This strain has a Bcf group A allele and a Stf group A1 allele. After inducing expression, we detected a large band of about 22 to 23 kDa from heat extractions of *E. coli* for both fimbriae (data not shown). Fimbriae were seen on bacterial surfaces by transmission electron microscopy for Stf (Fig. 4A) and Bcf (Fig. 4E) but not on bacteria containing empty vector pBAD33 (Fig. 4C) or pHSG576 (Fig. 4G) after addition of the corresponding inducer. Anti-Stf or -Bcf antisera were prepared against isolated fimbriae that were adsorbed against nonfimbriated bacteria. The specificity of the adsorbed antisera was confirmed by enzyme-linked immunosorbent assay (ELISA) as described in Materials and Methods, with comparable cross-reactive titers for the Bcf or Stf allelic adhesin variants. Electron microscopy of fimbriated bacteria confirmed that the fimbriae seen on bacterial surfaces were indeed Stf or Bcf fimbriae, using adsorbed fimbria-specific sera for immunogold labeling (Fig. 4B and F) and isogenic bacteria with corresponding empty vectors as negative controls (Fig. 4D and G), as described in Materials and Methods.

### Bcf and Stf alleles mediate preferential bacterial binding in a host species-dependent manner.

**FIG 5:**
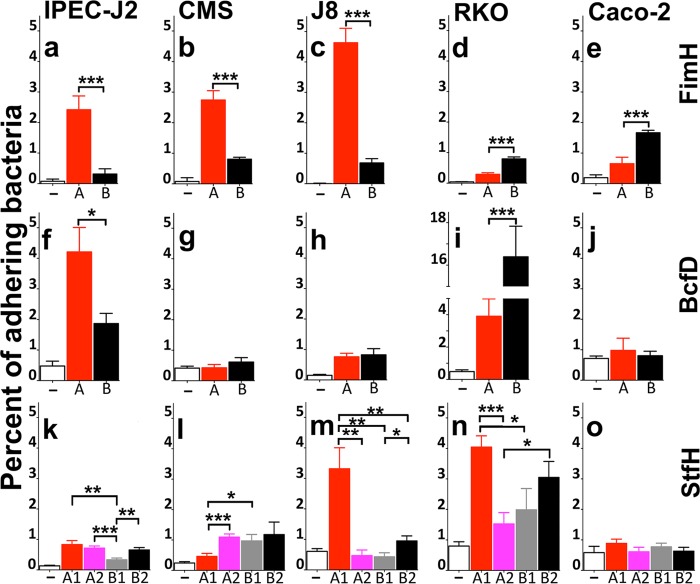
Bacterial adhesion to host-specific intestinal epithelial cells. The binding of recombinant *E. coli* AAEC189 expressing *S*. Newport Fim, Bcf, or Stf fimbriae with their different adhesin alleles, respectively, FimH (a to e), BcfD (f to j), or StfH (k to o), was determined with porcine (IPEC-J2), bovine (CMS and J8), and human (RKO and Caco-2) intestinal epithelial cell lines. The histograms indicate the percentages of adherent bacteria (CFU of the cell-associated bacteria divided by CFU of the inoculum). Results are the means of the results of three independent assays done in triplicate, with error bars representing standard errors. Asterisks above each bar represent significant differences (unpaired *t* tests) between percentages of bound bacteria with allele A or A1 (red bars), allele A2 (pink bars), allele B or B2 (black bars) or allele B1 (gray bars) for the respective adhesins, as shown on the right side (*, *P* < 0.05; **, *P* < 0.01; ***, *P* < 0.001). Nonfimbriated *E. coli* AAEC189 pAZ37 (Fim), pBAD33 (Stf), or pHSG-576 (Bcf) was used as a background binding control (open bars) to determine statistically significant adhesion, as mentioned in Results. The FimH data are from Yue et al. (19).

We previously showed that FimH alleles from various *Salmonella* serovars bound best to cells from the hosts that were the source of the isolates (19, 32). Even though host-species preferential binding was greatest for host-adapted serovars, corresponding results were obtained for a few tested broad-spectrum serovars, including Newport. Bovine-associated group A FimH from *S. enterica* serovar Newport mediated bacterial adhesion to bovine and porcine cells the best, whereas human-associated group B FimH was best at directing binding to human cells (19, 20) (Fig. 5a to e) (Table S3A). To determine whether the Bcf and Stf fimbrial adhesins also govern allele-dependent preferential binding to certain host species, and particularly to allele-associated hosts, adhesion to human, bovine, and porcine intestinal epithelial cells was investigated with *E. coli* engineered to express fimbriae with different adhesin alleles. Comparing levels of Bcf-mediated bacterial binding, bacteria that expressed these fimbriae with any adhesin allele bound significantly better to human RKO and porcine IPEC-J2 cells than the nonfimbriated control (Fig. 5f and i; *P* < 0.01) (Table S3B). The human Caco-2 cell line was not recognized by Bcf, possibly due to a lack of specific receptor expression in this cell line under the growth conditions used. Bacteria expressing allele A also bound significantly better to IPEC-J2 cells than bacteria with allele B (*P* < 0.05), while the result was reversed for the RKO cells (*P* < 0.001). Even though BcfD alleles showed much lower affinities for bovine cells, both alleles still mediated significant binding to bovine J8 cells (Fig. 5h; *P* < 0.05) (Table S3B). Thus, like the FimH adhesin, BcfD alleles modulate differential binding for various host intestinal epithelial cells. These results also uncovered a causal relationship that is in agreement with the previously suggested BcfD allele-host species association (20).

10.1128/mSphere.00066-17.6 TABLE S3  Statistical data (*, *P* < 0.05; **, P < 0.01; ***, *P* < 0.001 [unpaired *t* tests]) for the numbers of adherent strains with different FimH (A), BcfD (B), or StfH (C) alleles that bind to each intestinal epithelial cell line (Ø, nonfimbriated empty vector control strain). Download TABLE S3, PDF file, 0.04 MB. Copyright © 2017 De Masi et al. 2017 De Masi et al. This content is distributed under the terms of the Creative Commons Attribution 4.0 International license.

The bacterial adherence pattern for the StfH alleles was different from the patterns seen with FimH or BcfD alleles (Fig. 5k to o) (Table S3C). While bacteria with the StfH B2 allele bound significantly (*P* < 0.001) and preferentially to human RKO cells, bacteria with the A1 allele adhered significantly not only to the bovine J8 cells but also to the RKO cell line (*P* < 0.001). In contrast, bacterial binding to the other cell lines was noticeably weaker, though significant, for the StfH A2 and B alleles with the bovine CMS cells and for all the alleles with the porcine IPEC-J2 cells. As with the Bcf fimbriae, the Caco-2 cells did not present a binding target for Stf-fimbriated bacteria. The opposite adhesive properties of the A1 and A2 alleles, which switch their preferences between bovine epithelial cells of jejunal and colonic origin, hinted at an additional level of receptor selectivity that is tissue dependent. Finally, in contrast to the inhibition of Fim-mediated bacterial binding to host cells by mannose (19), the adhesion of bacteria expressing Bcf or Stf fimbriae to cells was “mannose-resistant” (Fig. S3).

10.1128/mSphere.00066-17.3 FIG S3  Bacterial adhesion inhibition for Fim, Bcf, or Stf fimbriated *E. coli* and IPEC-J2 or RKO cells. Binding of recombinant *E. coli* AAEC189 expressing *S*. Newport Fim, Bcf, or Stf fimbriae with their different adhesin alleles, including FimH A and B alleles, BcfD A and B alleles, and StfH A1 and B1 alleles. ΔFimH, ΔBcfD, and ΔStfH are corresponding empty vector controls. White columns indicate percentages of bacteria adhering to the corresponding intestinal epithelial cells; black columns indicate percentages of bacteria adhering in the presence of 50 mM methyl α-d-mannopyranoside. Data are means of results of triplicate assays (± standard errors of the means [SEM]), repeated independently three times. *P* values were calculated by using a one-sided unpaired *t* test (*, *P* < 0.05; ns, *P* > 0.05). Download FIG S3, PDF file, 0.1 MB. Copyright © 2017 De Masi et al. 2017 De Masi et al. This content is distributed under the terms of the Creative Commons Attribution 4.0 International license.

## DISCUSSION

This study identified surface-exposed organelles following expression of *Salmonella* fimbrial gene clusters *bcf* and *stf* and characterized differential allele-specific functions of their adhesins. These adhesins and their allelic variants allowed bacteria to discriminate not only between intestinal epithelial cells from different host species but also between enterocytes and colonocytes, in support of a model of preferential allele-directed bacterial colonization of specific hosts and distinct intestinal segments.

Our previous comparisons of 90 *Salmonella* genomes highlighted 35 chaperone-usher gene clusters, or 5 to 14 clusters per strain, each including an open reading frame for a known or predicted adhesin with a variety of nonsynonymous single nucleotide polymorphisms (nsSNPs) (11). Some adhesin alleles were found to be associated with host species that served as the source of the respective strains (19, 20). A biological explanation for these associations was sought with the FimH adhesin (19). Type 1 fimbriae expressing FimH from bacteria isolated from a specific host best mediated bacterial adhesion to intestinal epithelial cells from the corresponding host species. This affinity was most impressive for the FimH from host-restricted or -adapted *Salmonella* serovars, although functional association also matched specific associations with FimH from broad-host-range strains. A larger proportion of S. Typhimurium and *S*. Newport strains isolated from humans encoded a FimH allele that promoted better bacterial binding to human than to bovine intestinal epithelial cells, while the reverse result was obtained with bovine isolates. Since the two *S*. Newport groups with FimH alleles responsible for the opposite binding properties had previously been shown to also encode separate alleles for the predicted adhesins of the Bcf and Stf fimbriae (20), we characterized here the potential participation of these adhesin alleles for potential host specificity.

In a first step, we evaluated the major alleles of the FimH, BcfD, and StfH fimbrial adhesins for 262 *S*. Newport strains and confirmed the previous description of two allelic groups (labeled A and B) for each adhesin (20). The sequences of the FimH groups were published previously (19, 20). Bcf alleles differed by only 3 amino acids, whereas more differences separated StfH allelic groups A and B, which could each be further divided into two subgroups (subgroup A1 and A2 and subgroup B1 and B2). While the N-terminal half of StfH differed markedly between its major groups, only one or three different amino acids determined the two subgroups. Other proteins encoded by the Bcf and Stf gene clusters showed no variation (data not shown).

Our previous work associated FimH group A allele with bovine strains, and most strains with FimH allele A were associated with A alleles for BcfD and StfH (20). However, due to the low number of nonbovine strains that were pooled for comparisons, the results from allelic group A of BcfD and StfH suggested an associative trend for the bovine isolates without reaching statistical significance. Here, additional *S*. Newport sequence data were collected to compare allelic variants of the three adhesins for 262 strains isolated from different sources. The vast majority (90% to 96%) of the bovine isolates had a *fimH* group A, *bcfD* group A, and *stfH* group A allele, while 75% to 81% of the porcine isolates had these alleles. Isolates from avian and equine hosts had approximately the same numbers of each allelic group for the three adhesins, and while the FimH and BcfD allelic groups of human isolates had similar distributions, 76% contained a *stfH* B allele, closely mirroring the percentage found in environmental isolates (70%). Based on our previous findings for the FimH adhesin for which a causal relationship for the detected host association of alleles was confirmed by binding assays (19), the detected associations for BcfD and StfH alleles with distinct hosts opened the possibility that these alleles further contribute to preferential host-specific binding.

The Bcf and Stf fimbriae are not expressed by *Salmonella in vitro*. Thus, to confirm a functional relationship for the alleles of the predicted BcfD and StfH adhesins, we used various cloning approaches to construct bacteria capable of producing these fimbriae. The best results were obtained by cloning the Bcf gene cluster into a low-copy-number plasmid and the Stf gene cluster into a medium-copy-number plasmid with an inducible promoter. Fimbria-like structures were visualized on bacterial surfaces with both constructs, and their identities were confirmed by immune electron microscopy. Bacteria expressing the Bcf fimbriae bound well to a porcine jejunocyte and the human RKO colonic cell line, although the BcfD alleles presented opposite host preferences, with the A allele mediating stronger binding to the porcine cell and the B allele mediating stronger binding to the human cell. Similarly, the stfH alleles mediated differential levels of binding to various cell lines. Bacteria expressing fimbriae with the stfH allele B2 bound significantly better than the negative control to human RKO cells, while allele A1 mediated significant better adhesion to bovine jejunocytes and RKO cells. These results also strongly supported the predicted identification of BcfD and StfH as the adhesins of their respective fimbrial structures, although the possibility of indirect effects on other adhesive molecules cannot be fully excluded at this time. Ironically, Bcf, originally identified as a bovine colonization factor, bound the least to the bovine enterocytes, albeit it still bound significantly to the jejunocytes. Alleles with better affinities for bovine cells might exist in serovars other than Newport, including Typhimurium, the serovar studied when Bcf was first described.

The allele-determined preferential adhesion profiles indicated that amino acids residues 72, 289, and/or 300 in BcfD were critical for specificity with respect to human or porcine cells. One or more of these residues might be part of the binding pockets on BcfD or might be required for receptor-specific tertiary conformation of this adhesin. In addition, positions 108, 109, and/or 202 of StfH played a role in the binding to bovine and human cells, since their substitutions in A2 alleles greatly reduced adhesion. Only six strains had the StfH A2 allele, suggesting that they might be less successful animal colonizers than other strains; it remains possible that this allele binds to cells from animals that were not included in this study. Members of allelic group B1 and B2 are distinguished by a substitution of an asparagine for an aspartic acid at position 127, resulting in a residue switch with position 128 for the B2 allele compared to the two A alleles and improving the properties of adhesion of this allele to RKO and J8 cells. Taking the data together, a few unique substitutions in BcfD and StfH alleles contribute by themselves to the quantitative and specific binding properties of the bacteria expressing the corresponding fimbriae. Fimbriated bacteria did not always bind similarly to cells from the same host, highlighting variable receptor presentation in hosts, likely because the cells originated from different intestinal segments, albeit the possibility of individual differences in one host species cannot be excluded. Moreover, some of the cells were transformed cells and might have had an altered receptor profile on their surface. Nevertheless, our findings on the differential binding affinities of *S*. Newport FimH, BcfD, and StfH as investigated with a limited number of cells from a few host species were consistent with the participation of adhesin alleles in host adaptation, as previously observed with S. Typhimurium FimH (19).

Central to the lifestyle of *S. enterica* is the assortment of alleles among various major adhesins that should have a determining influence on the optimal sites of bacterial colonization, namely, the animal host(s) in which survival, settling, multiplication, and transmission are favored (33). Indeed, a comparison of the three adhesin alleles for each isolate according to host origin (Fig. 3; see also Fig. S1 in the supplemental material) and host-determined binding properties (Fig. 5) indicated that most of the isolates from one host carry at least two allelic variants that adhere significantly to intestinal epithelial cells from that host. More than 80% of the porcine isolates had a *fimH* A allele with a *bcfD* A or B allele, while 88% of the bovine isolates had a *fimH* A allele and a *stfH* A1 allele, both allelic sets coding for adhesins that bind to an intestinal epithelial cells from the corresponding host. Similarly, 97% of the human isolates possessed at least two adhesin variants that bind significantly to a human cell.

In contrast to the porcine and bovine isolates, which were characterized as having predominantly a FimH/BcfD/StfH set of A/A/A1 alleles (Fig. 3), the human isolates were split roughly 50/50 for *bcfD* and *fimH* A and B alleles and 30/70 for *stfH* (Fig. S1) with three to four major sets of alleles (Fig. 3). These results were in agreement with a previous phylogenetic study by Sangal et al. that identified two major *S*. Newport lineages in North America (34), with an “animal” lineage (designated Newport-II) corresponding to strains with the FimH/BcfD/StfH A/A/A1 alleles in this study and North American human isolates (Newport-III) relating to strains with the FimH/BcfD/StfH B/B/B1 alleles (see Table S1 in the supplemental material). A minor third lineage (Newport-I) was also represented by a few human isolates, mainly from Europe, in our study.

The distribution of the three adhesin alleles in human isolates is consistent with their binding affinities for human cells and with various sources of human infections with *S*. Newport. Indeed, each of the other hosts had large (~20% or more) proportions of isolates with fimbriae that bound well to human cells. Thus, a *S*. Newport strain isolated from a human patient with a *fimH bcfD stfH* B/B/B1 set of alleles would more likely have originated from an avian, equine, or environmental (e.g., contaminated vegetable) source, while a human isolate with an A/A/A1 set of alleles would probably have been from a bovine or porcine source. By analogy, the source of the human isolates with the second-most-frequent set of adhesin alleles (A/A/B2) might be pigs, since such strains were found only in these animals, albeit in small numbers.

Given the number of fimbrial gene clusters among the various *Salmonella* serovars (11), it is probable that many of these genes encode a functional adhesive structure with differential host and tissue specificities. Alleles of nonfimbrial adhesins might add to a complicated interplay of adhesin sets that contribute to host-specific colonization. However, a selected few major adhesins might already demonstrate detectable phenotypic associations. This study focused on the three fimbrial adhesins of *S*. Newport previously found to have alleles with significant or potential associations with at least one host species (20). We first demonstrated that two putative fimbrial gene clusters of *S*. Newport, Bcf and Stf, encode effectively fimbrial structures. The analysis of 262 strains identified an association between alleles of the predicted adhesins FimH, BcfD, and StfH and the specific host species from which the strains were isolated. Moreover, a cause and effect relationship was established by the detection of preferential binding by specific adhesin alleles to intestinal epithelial cells from corresponding hosts. Distributions of these adhesin alleles were markedly different among isolates from diverse host groups, and isolates carrying genes for at least two adhesin alleles binding preferentially to one host species were also isolated more frequently from this host. These discovered allele-host associations might serve in the investigation of *S*. Newport outbreaks.

In summary, allelic variability leads to different combinations of adhesins with multiple host specificities. Some allelic combinations contribute to maintain the bacteria in a large number of host species. However, convergence of several adhesin alleles for a few or one host species allows successful clonal expansion in the corresponding specific host(s). Such clones model an early evolutionary step toward host adaptation as found with various *Salmonella* serovars.

## MATERIALS AND METHODS

### Bacterial strains, growth conditions, and plasmid constructions.

All bacterial strains and plasmids used for experiments are listed in Table S4 in the supplemental material, whereas PCR primers are described in Table S5. Unless otherwise indicated, bacteria were grown in LB with appropriate antibiotics for plasmid maintenance. The entire *bcf* and *stf* gene clusters, from *bcfA* through *bcfG* or *bcfH* and from *stfA* through *stfH* from strain SL254, were amplified by PCR. The *bcf* gene cluster was cloned into the BamHI-HindIII sites of low-copy-number vector pHSG576 to create vector pLDHSG-Bcf-S, containing *bcfA* through *bcfG*, or vector pLDHSG-Bcf-L, containing *bcfA* through *bcfH*. The *stf* gene cluster was cloned into the multiple-cloning region of the pBAD33 expression vector to create plasmid pLDBAD-Stf. *E. coli* SE5000 or AAEC189 was transformed by electroporation. Overnight LB cultures of *E. coli* containing pLDHSG-Bcf-S or pLDBAD-Stf were diluted 1:50 in LB and incubated for 2 h at 37°C. Bcf and Stf fimbrial expression was induced with 1 mM IPTG (isopropyl-β-d-thiogalactopyranoside) and 0.02% arabinose, respectively, and bacteria were grown for an additional 3 h with minimal shaking. For allelic substitution, *bcfD* was deleted from pLDHSG-Bcf-S by inverse PCR, *bcfD* from strain SL317 was amplified by PCR, and both fragments were assembled using a Gibson Cloning reaction (NEB [New England Biolabs], Ipswich, MA). To replace *stfH* of pLDBAD-Stf with various alleles, the plasmid was first digested with SpeI and HindIII (NEB) and the linear fragment lacking *stfH* DNA was isolated by agarose gel electrophoresis. PCR-amplified *stfH* alleles from different strains were then cloned into the linearized fragment using a Gibson assembly system (NEB). All plasmid constructs were checked for accuracy by DNA sequencing. S. Typhimurium AJB4 *bcfC*::miniTn*5* was obtained by generalized transduction with P22 HT *int* using STN35 as the donor strain.

10.1128/mSphere.00066-17.7 TABLE S4  Strains and plasmids used in this study. Download TABLE S4, PDF file, 0.03 MB. Copyright © 2017 De Masi et al. 2017 De Masi et al. This content is distributed under the terms of the Creative Commons Attribution 4.0 International license.

10.1128/mSphere.00066-17.8 TABLE S5  Primers used for PCR in this study. Download TABLE S5, PDF file, 0.02 MB. Copyright © 2017 De Masi et al. 2017 De Masi et al. This content is distributed under the terms of the Creative Commons Attribution 4.0 International license.

### Identification of FimH, BcfD, and StfH alleles in *Salmonella* Newport genomes.

Sequence data were obtained from 281 different strains of *S*. Newport that included bovine, equine, porcine, avian, human, and environmental isolates. First, the *S*. Newport *fimH*, *bcfD* and *stfH* sequences were collected from 23 strains identified in our previous work (11, 20) and from 31 complete genomes in GenBank. Second, raw genomic data of 227 strains were collected from GenomeTrakr (35) and assembled using Velvet (36). The genomic sequences were aligned with BioEdit (Ibis Biosciences, Carslbad, CA), and *bcfD*, *stfH*, and *fimH* alleles were identified using BLAST. Predicted protein sequences were aligned with ClustalW (MegAlign; DNAStar, Madison, WI). Of the 227 strains assembled from raw data, 19 were not used in this study, since sequence information was missing or incomplete for 11 strains and 8 strains had minor adhesin alleles (slight variations for the group A and/or B alleles), a number that was too low for significant evaluation. Thus, major adhesin alleles were obtained for a total of 262 strains, all collected over a span of 19 years (1997 to 2015) and from geographically distinct locations (Table S1). In addition, sequence types and phylogenetic lineages (34) were determined for the strains with available genomic data by using SRST2 (37) (Table S1).

### Preparation of fimbriae and antibodies.

Production of Bcf or Stf fimbriae was induced in *E. coli* SE5000 (500-ml cultures), as described above. Cells were pelleted by centrifugation, resuspended in 10 ml suspension buffer (75 mM NaCl, 0.5 mM Tris-Cl, pH 7.5), and incubated for 30 min at 50°C for Bcf or 60°C for Stf. Samples were centrifuged for 2 min at 16,000 × *g*, bacterial pellets were discarded, and supernatants were spun a second time. Ammonium sulfate was added to the supernatants to reach a final concentration of 25% (wt/vol). After an overnight incubation at 4°C, samples were centrifuged for 15 min at 16,000 × *g*. Ammonium sulfate was added to the supernatants at final concentrations of 50% for Bcf and 40% for Stf, and the mixtures were incubated overnight at 4°C. Solutions were centrifuged for 15 min at 16,000 × *g*, and the pellets were suspended in 1 ml of phosphate-buffered saline (PBS) for overnight dialysis at 4°C, using 3,000 molecular weight cutoff (MWCO) dialysis tubing (Spectrum Laboratories, Inc., Irving, TX). The presence of fimbriae was confirmed by SDS-PAGE and Coomassie blue staining. The recombinant protein preparations were used for the generation of specific anti-Bcf and -Stf antibodies in rabbits (Cocalico Biologicals, Inc., Reamstown, PA). The antisera were adsorbed three times against S. Typhimurium AJB4 *bcfC*::miniTn*5* or AAEC189 containing pBAD33 for the anti-Bcf or anti-Stf antibody, respectively (38). For this, 1 ml antisera with 0.02% sodium azide was incubated for 18 h at 4°C with bacterial pellets from 10-ml cultures grown overnight; after three adsorption cycles, the sera were filtered (0.02-μm-pore-size filter) before use. ELISA titers were 7 × 10^−3^ for Bcf and 7 × 10^−4^ for Stf using fimbriated bacteria as the antigen and isogenic nonfimbriated bacteria as negative controls. The bacterial constructs with the various allelic adhesins expressed comparable levels of the corresponding Bcf or Stf fimbriae on the surface of *E. coli* after induction, as confirmed by ELISA (not shown) (19).

### Electron microscopy.

For transmission electron microscopy, AJB4 pLDHSG-Bcf-L and SE5000 pLDHSG-Bcf-S or SE5000 pLDBAD-Stf were grown and induced for fimbrial expression as described above. A 10-μl volume of cell culture was incubated for 10 min on a glow-discharged 400-mesh copper grid (EMS Sciences, Hatfield, PA) and then wicked dry. Grids were washed 6 times with 50-μl drops of distilled water, wicked dry, and stained for 5 min with 2% uranyl acetate. Grids were wicked dry again, washed with one drop of distilled water, and wicked dry before use. Grids were loaded onto a Tecnai T12 transmission electron microscope (FEI) containing a tungsten filament operating at 80 kV. Images magnified at ×10,000, ×15,000, and ×20,000 were taken with a Gatan US1000 (2 K ×2 K) charge-coupled-device (CCD) camera (Gatan, Pleasanton, CA). For immunogold electron microscopy, cells were grown and induced for fimbrial expression as described above and incubated with adsorbed anti-Bcf (10^−2^ dilution) or anti-Stf (2 × 10^−3^ dilution) serum overnight at 4°C. The bacteria were centrifuged (200 × *g*, 30 s) to carefully remove supernatants and mixed with 500 μl PBS before incubation on ice was performed for 30 min. Supernatants of settled bacteria were removed, and the bacteria were mixed again with 500 μl PBS before incubation on ice was performed for 20 min. This was repeated once more before anti-rabbit antibodies conjugated with 10-nm-diameter gold particles (Sigma-Aldrich Corp., St. Louis, MO) were added to reach a final concentration of 5 × 10^−2^. After being maintained overnight at 4°C, the bacteria were centrifuged and washed as described for the primary antibody, placed on 400-mesh copper grids (10 μl), and fixed with 4% paraformaldehyde for 20 min. Grids were washed 6 times with distilled H_2_O (dH_2_O) (50-μl drops), stained for 5 min with 2% uranyl acetate, and washed an additional time with dH_2_O before examination by electron microscopy.

### Adherence assay.

The human Caco-2 (ATCC HTB-37) and RKO (ATCC CRL2577) colonic epithelial cell lines, porcine IPEC-J2 (DSMZ ACC 701) jejunal epithelial cells, and bovine colonic CMS and jejunal J8 epithelial cells (19, 39, 40) were all grown to 80% confluence in T-75 culture flasks at 37°C (5 to 10% CO_2_). Cells were detached with 0.25% trypsin and used to seed 24-well plates at a concentration of 1 × 10^5^ cells/ml and incubated overnight. Wells were washed 3 times with sterile PBS prior to the addition of bacteria. AAEC 189 cells were grown to express Bcf and Stf fimbriae as described above, diluted 1.4 × 10^−1^ in Dulbecco’s modified Eagle medium/nutrient mixture F-12 (DMEM-F12) (Life Technologies, Inc.), and loaded at a multiplicity of infection (MOI) of 100 onto 24-well plates (3 replicates per bacterium). After 1 h of incubation at 37°C, wells were washed 3 times with sterile PBS to remove unassociated bacteria. PBS with 0.1% Triton X-100 (100 μl) was added to each well and left for 5 to 10 min at 4°C to release the cell-associated bacteria. Bacterial counts (CFU) were determined by standard techniques and used to determine the percentage of adherent bacteria (CFU of the cell-associated bacteria divided by CFU of the inoculum).
