# Supplementary material for: Cooperation of Adhesin Alleles in Salmonella-Host Tropism
Source: mSphere. 2017 Mar 8;2(2):e00066-17. doi: 10.1128/mSphere.00066-17 (PMC5343171; doi:10.1128/mSphere.00066-17)
Supplement: TABLE S1 [file sph002172250st1.pdf]

Table S1. *Salmonella* Newport strains and corresponding metadata

Strains	Host_Group	Place of Isolation	Continents	Year	Lineage*	FimH	StfH	BcfD	Accession
**3759**	Human	UK:London	Europe	2014	II	A	B2	A	SRR1966608
**5871**	Human	UK:North of England	Europe	2014	II	A	B2	A	SRR1966910
**5922**	Human	UK:North of England	Europe	2014	II	A	B2	A	SRR1965892
**5927**	Human	UK:South of England	Europe	2014	II	A	B2	A	SRR1968617
**6443**	Human	UK:North of England	Europe	2014	II	A	B2	A	SRR1967603
**6444**	Human	UK:Wales	Europe	2014	III	B	B1	B	SRR1968167
**9133**	Human	UK:London	Europe	2014	II	A	A1	A	SRR1967210
**78677**	Human	UK:North of England	Europe	2014	II	A	A1	A	SRR1963352
**78687**	Human	UK:London	Europe	2014	II	A	B2	A	SRR1963447
**79051**	Human	UK:South of England	Europe	2015	III	B	B1	B	SRR1963296
**80366**	Human	UK:Midlands and East of England	Europe	2015	II	A	B2	A	SRR1962455
**83328**	Human	UK:North of England	Europe	2015	III	B	B1	B	SRR1968384
**83347**	Human	UK:North of England	Europe	2015	II	A	B2	A	SRR1969769
**83350**	Human	UK	Europe	2015	III	B	B1	B	SRR1969117
**85686**	Human	UK:South of England	Europe	2014	II	A	A1	A	SRR1966335
**86886**	Human	UK:South of England	Europe	2015	III	B	B1	B	SRR1962455
**89100**	Human	UK:North of England	Europe	2015	II	A	A1	A	SRR1966341
**89103**	Human	UK:South of England	Europe	2015	I	B	B1	B	SRR1969927
**89990**	Human	UK:South of England	Europe	2015	II	A	A1	A	SRR1969616
**90000**	Human	UK:Midlands and East of England	Europe	2015	II	A	A1	A	SRR1965362
**93543**	Human	UK:London	Europe	2015	II	A	A1	A	SRR1967791
**94249**	Human	UK:Midlands and East of England	Europe	2015	I	B	B2	B	SRR1965901
**94271**	Human	UK:North of England	Europe	2015	II	A	B2	A	SRR1966159

**WAPHL_SAL_A00644**	Human	USA:WA	North America	2014	II	A	A1	A	SRX745794
**WAPHL_SAL_A00645**	Human	USA:WA	North America	2014	III	B	B1	B	SRX745423
**WAPHL_SAL_A00646**	Human	USA:WA	North America	2014	III	B	B1	B	SRX796195
**WAPHL_SAL_A00647**	Human	USA:WA	North America	2014	II	A	A1	A	SRX745424
**WAPHL_SAL_A00648**	Human	USA:WA	North America	2014	III	B	B1	B	SRX749304
**WAPHL_SAL_A00649**	Human	USA:WA	North America	2014	III	B	B1	B	SRX749305
**WAPHL_SAL_A00650**	Human	USA:WA	North America	2014	II	A	B2	A	SRX749298
**WAPHL_SAL_A00651**	Human	USA:WA	North America	2014	I	B	B1	B	SRX749308
**CDC2010K-2159**	Human	USA	North America	2010	II	A	B1	A	CP007559.1
**SL317**	Human	USA:MN	North America	2002	III	B	B1	B	ABEW00000000
**SL254**	Human	USA:MN	North America	2000	II	A	A1	A	CP001113
**DC_10_446**	Human	USA:DC	North America	2010	III	B	B1	B	SRR648847;SRR643617
**DC_10_449**	Human	USA:DC	North America	2010	III	B	B1	B	SRR648848;SRR643618
**DC_10_450**	Human	USA:DC	North America	2010	III	B	B1	B	SRR648849;SRR643619
**FAR0182**	Human	USA:TX	North	2010	III	B	B2	B	SRX554411

			America						
**FCC0004**	Human	USA:NM	North America	2010	III	B	B2	B	SRX380210
**FCC0078**	Human	USA:NM	North America	2010	III	B	B2	B	SRX404467
**H123940625**	Human	UK:London	Europe	2012	III	B	B1	B	SRR1635122
**MA_10EN1469**	Human	USA:MA	North America		III	B	B1	B	SRR648846;SRR643616
**NY_swgs1512**	Human	USA:NY	North America	2014	III	B	B1	B	SRX745801
**SH111077**	Human	China:Shanghai	Asia	2011	II	A	A1	A	AOGJ00000000
**VA_R100804798**	Human	USA:VA	North America		III	B	B1	B	SRR648844;SRR643614
**VA_R100808502**	Human	USA:VA	North America		III	B	B1	B	SRR648845;SRR643615
**CFSAN000841**	Env	USA:VA	North America	2008	III	B	B1	B	APGQ00000000
**CFSAN000843**	Env	USA:VA	North America	2008	III	B	B1	B	APGR00000000
**CFSAN000847**	Env	USA:VA	North America	2008	III	B	B1	B	APGS00000000
**CFSAN000852**	Env	USA:VA	North America	2008	III	B	B1	B	APGT00000000
**CFSAN000854**	Env	USA:VA	North America	2008	III	B	B1	B	APGU00000000
**CFSAN000857**	Env	USA:VA	North America	2008	III	B	B1	B	APGV00000000
**CFSAN0009**	Env	USA:VA	North	2006	III	B	B1	B	APHC00000000

**27**			America						
**CFSAN000928**	Env	USA:VA	North America	2006	III	B	B1	B	APHD00000000
**CFSAN000947**	Env	USA:VA	North America	2007	III	B	B1	B	APHF00000000
**CFSAN001243**	Env	USA:VA	North America	2010	III	B	B1	B	APHG00000000
**CFSAN002005**	Env	USA:IN	North America	2012	III	B	B1	B	SRR1505074
**CFSAN002006**	Env	USA:IN	North America	2012	III	B	B1	B	SRR1505063
**CFSAN002011**	Env	USA:IN	North America	2012	III	B	B1	B	SRR1505054
**CFSAN002012**	Env	USA:IN	North America	2012	III	B	B1	B	SRR1505008
**CFSAN002013**	Env	USA:IN	North America	2012	III	B	B1	B	SRR1505064
**CFSAN002014**	Env	USA:IN	North America	2012	III	B	B1	B	SRR1504997
**CFSAN002016**	Env	USA:IN	North America	2012	III	B	B1	B	SRR1504998
**CFSAN002018**	Env	USA:IN	North America	2012	III	B	B1	B	SRR1505075
**CFSAN014125**	Env	Argentina	South America	2003	II	A	A1	A	SRR1615949
**CFSAN024485**	Env	Mexico:Sinaloa	North America	2010	II	A	A1	A	SRR2015658
**CFSAN024492**	Env	Mexico:Sinaloa	North America	2011	II	A	A1	A	SRR2054263

**CFSAN024503**	Env	Mexico:Sinaloa	North America	2011	II	A	A1	A	SRR2053322
**CFSAN024504**	Env	Mexico:Sinaloa	North America	2011	III	B	B1	B	SRR2053337
**CFSAN024507**	Env	Mexico:Sinaloa	North America	2011	II	A	A1	A	SRR2054279
**CFSAN024508**	Env	Mexico:Sinaloa	North America	2011	II	A	A1	A	SRR2054184
**CFSAN024510**	Env	Mexico:Sinaloa	North America	2011	II	A	A1	A	SRR2053332
**CFSAN024512**	Env	Mexico:Sinaloa	North America	2011	III	B	B2	B	SRR2054113
**CFSAN024530**	Env	Mexico:Sinaloa	North America	2011	II	A	A1	A	SRR2054211
**CFSAN024536**	Env	Mexico:Sinaloa	North America	2011	II	A	A1	A	SRR2054274
**CFSAN024537**	Env	Mexico:Sinaloa	North America	2011	II	A	A1	A	SRR2053348
**CFSAN024538**	Env	Mexico:Sinaloa	North America	2012	II	A	A1	A	SRR2053317
**CFSAN024546**	Env	Mexico:Sinaloa	North America	2012	II	A	A1	A	SRR2054269
**CFSAN024554**	Env	Mexico:Sinaloa	North America	2012	II	A	A1	A	SRR2054241
**CFSAN024555**	Env	Mexico:Sinaloa	North America	2012	II	A	A1	A	SRR2054114
**CFSAN024567**	Env	Mexico:Sinaloa	North America	2012	III	B	B1	B	SRR2054270
**CFSAN0245**	Env	Mexico:Sinaloa	North	2012	III	B	B1	B	SRR2054185

**74**			America						
**CFSAN024575**	Env	Mexico:Sinaloa	North America	2012	III	B	B1	B	SRR2054202
**A182RVB**	Env	USA:VA	North America	2007	III	B	B1	B	SRR648852
**FAR0008**	Env	Mexico	North America	2010	III	B	B1	B	SRX380200
**FAR0040**	Env	Mexico	North America	2010	III	B	B1	B	SRX462117
**FAR0115**	Env	India	Asia	2013	II	A	B1	A	SRX528122
**FCC0219**	Env	Mexico	North America	2008	III	B	B1	B	SRX734171
**FDA00001361**	Env	Mexico	North America	2009	III	B	B2	B	SRX814817
**Levine11_3**	Env	USA:VA	North America		III	B	B1	B	AUQQ01
**Levine11_4**	Env	USA:VA	North America		III	B	B1	B	AUQP01
**Pond080_2TTA**	Env	USA:VA	North America	2006	III	B	B1	B	SRR648853
**PRS_2010_0624**	Env	USA:VA	North America	2010	III	B	B1	B	SRR648858
**CVM21538**	Avian	USA:GA	North America		II	A	A1	A	AHTV00000000
**CVM21539**	Avian	USA:MO	North America		III	B	B1	B	AHTL00000000
**CVM21559**	Avian	USA:CO	North America		III	B	B1	B	AHTO00000000
**CVM33953**	Avian	USA:MD	North	2003	III	B	B2	B	AHTM00000000

			America						
**CVMN18486**	Avian	USA:NM	North America	2008	II	A	A1	A	AHTY00000000
**S103RVX**	Avian	USA:VA	North America	2006	III	B	B2	B	SRR648854;SRR643620
**AZ_TG68156**	Avian	USA:NM	North America	2009	II	A	A1	A	SRX336189
**AZ_TG68216**	Avian	USA:NM	North America	2009	II	A	A1	A	SRX336193
**AZ_TG74216**	Avian	USA:CO	North America	2005	II	A	A1	A	SRX755933
**AZ_TG74220**	Avian	USA:CO	North America	2005	II	A	A1	A	SRX663092
**CFSAN024412**	Avian	Belgium	Europe	2010	I	B	B1	B	SRX754525,SRX869028
**CFSAN024413**	Avian	Belgium	Europe	2010	III	B	B1	B	SRX754520,SRX869039
**CFSAN024415**	Avian	Belgium	Europe	2013	I	A	B1	B	SRX754521,SRX869045
**CFSAN024417**	Avian	Belgium	Europe	2013	I	A	B1	B	SRX754531,SRX869030
**CFSAN026625**	Avian	Belgium	Europe	2011	II	A	A1	A	SRX845979
**CFSAN026626**	Avian	Belgium	Europe	2012	III	B	B1	B	SRX846013
**MDH_2013_00021**	Avian	USA:MN	North America	2007	II	A	A1	A	SRX476431
**MDH_2013_00024**	Avian	USA:MN	North America	2007	II	A	A1	A	SRX476718

**MDH_2013_ 00098**	Avian	USA:MN	North America	2009	III	B	B1	B	SRX475531
**MDH_2013_ 00171**	Avian	USA:MN	North America	2012	II	A	A1	A	SRX403842
**MDH_2013_ 00187**	Avian	USA:MN	North America	2012	III	B	B2	B	SRX390905
**MDH_2014_ 0087**	Avian	USA:MN	North America	2003	II	A	A1	A	SRX532276
**MDH_2014_ 0124**	Avian	USA:MN	North America	2005	II	A	A1	A	SRX523552
**MDH_2014_ 00340**	Avian	USA:MN	North America	2002	II	A	A1	A	SRX626716
**MOD1_Sal_ 917**	Avian	USA:PA	North America	2009	III	B	B1	B	SRX679128
**NY_22697**	Avian	USA:MD	North America	2002	II	A	A1	A	SRX652336
**NY_BAC090 0000179**	Avian	USA:NY	North America	2009	II	A	A1	A	SRX462115,SRX472503
**NY_swgs11 27**	Avian	USA:MN	North America	2008	II	A	A1	A	SRX529496
**NY_swgs11 38**	Avian	USA:MN	North America	2008	II	A	A1	A	SRX529482
**NY_swgs11 61**	Avian	USA:MN	North America	2010	II	A	A1	A	SRX529464
**VA_WGS_0 0342**	Avian	USA:VA	North America	2011	III	B	B1	B	SRX738208
**VA_WGS_0 0372**	Avian	USA:VA	North America	2012	III	B	B1	B	SRX765985
**VA_WGS_0**	Avian	USA:VA	North	2012	III	B	B1	B	SRX765976

**0373**			America						
**VA_WGS_0 0387**	Avian	USA:VA	North America	2012	III	B	B1	B	SRX796829
**VA_WGS_0 0388**	Avian	USA:VA	North America	2012	III	B	B2	B	SRX757337
**VA_WGS_0 0389**	Avian	USA:VA	North America	2012	III	B	B1	B	SRX757338
**WAPHL_SAL _A00473**	Avian	USA:GA	North America	2004	III	B	B2	B	SRX663130
**WAPHL_SAL _A00474**	Avian	USA:GA	North America	2004	III	B	B2	B	SRX663132
**WAPHL_SAL _A00607**	Avian	USA:CO	North America	2005	II	A	A1	A	SRX739105
**CFSAN0226 22**	Avian	USA:AR	North America		III	B	B2	B	SRR2016680
**CFSAN0226 33**	Avian	USA:MO	North America		II	A	A1	A	SRR2016694
**CFSAN0329 52**	Avian	USA:WA	North America	2012	II	A	A1	A	SRR2075194
**CFSAN0009 29**	Avian	USA:VA	North America	2006	III	B	B1	B	APHE00000000
**A-211-RVH**	Avian	USA:VA	North America	2007	III	B	B1	B	SRX1125264
**0106-441**	Avian	USA:PA	North America	2001	?**	B	A1	A	Yue et al., 2012
**212-156**	Avian	USA:PA	North America	2002	?**	A	A2	A	Yue et al., 2012
**CVM35188**	Equine	USA:TN	North America	2004	III	B	B1	B	AHTN00000000

**CVM35202**	Equine	USA:TN	North America	2004	III	B	B2	B	AHTS00000000
**0307_213**	Equine	USA:PA	North America	2003	?**	A	A1	A	Yue et al., 2012
**MDH_2013_00038**	Equine	USA:MN	North America	2008	II	A	A1	A	SRX468959
**MDH_2014_0040**	Equine	USA:MN	North America	2003	II	A	A1	A	SRX553325
**MDH_2014_00498**	Equine	USA:MN	North America	2003	II	A	A1	A	SRX700225
**MDH_2014_00591**	Equine	USA:MN	North America	2004	II	A	A1	A	SRX758480
**NY_swgs1145**	Equine	USA:MN	North America	2009	III	B	B1	B	SRX529453
**VA_WGS_00103**	Equine	USA:VA	North America	2006	II	A	A1	A	SRX476713
**VA_WGS_00104**	Equine	USA:VA	North America	2006	II	A	A1	A	SRX675737,SRX503024
**VA_WGS_00133**	Equine	USA:VA	North America	2006	III	B	B1	B	SRX515707
**CFSAN022621**	Equine	USA:TX	North America		III	B	B2	B	SRR2016685
**CVMN1543**	Equine	USA:GA	North America	2004	II	A	A1	A	AHTZ00000000
**0207-412**	Equine	USA:PA	North America	2002	?**	A	A1	A	Yue et al., 2012
**1110-135**	Equine	USA:PA	North America	2011	?**	B	B1	B	Yue et al., 2012
**9711-223**	Equine	USA:PA	North	1997	?**	B	B2	B	Yue et al., 2012

			America						
**9809-140**	Equine	USA:PA	North America	1998	?**	B	B1	B	Yue et al., 2012
**CVM21550**	Porcine	USA:TX	North America		II	A	A1	A	AHTT00000000
**AZ_TG6847 1**	Porcine	USA:TX	North America	2012	III	B	B2	B	SRX391900
**CVM4176**	Porcine	USA:CA	North America		II	A	A1	A	AHUG00000000
**FSIS150280 7**	Porcine	USA:TN	North America	2015	II	A	B2	A	SRR2421548
**MDH_2013_ 00034**	Porcine	USA:MN	North America	2008	II	A	A1	A	SRX468908
**MDH_2013_ 00125**	Porcine	USA:MN	North America	2010	II	A	A1	A	SRX415318
**MDH_2013_ 00135**	Porcine	USA:MN	North America	2011	III	B	B1	B	SRX472502
**MDH_2013_ 00163**	Porcine	USA:MN	North America	2012	II	A	A1	A	SRX468962
**MDH_2013_ 00184**	Porcine	USA:MN	North America	2012	II	A	B2	A	SRX390794
**MDH_2014_ 00295**	Porcine	USA:MN	North America	2002	II	A	A1	A	SRX592874
**MDH_2014_ 00385**	Porcine	USA:MN	North America	2003	II	A	A1	A	SRX672461
**MDH_2014_ 00547**	Porcine	USA:MN	North America	2003	II	A	A1	A	SRX722089
**MDH_2014_ 00598**	Porcine	USA:MN	North America	2004	II	A	A1	A	SRX758466

**MDH_2014_ 00601**	Porcine	USA:MN	North America	2004	II	A	A1	A	SRX769773
**MDH_2014_ 00619**	Porcine	USA:MN	North America	2004	II	A	A1	A	SRX799301
**MDH_2014_ 00628**	Porcine	USA:MN	North America	2005	II	A	A1	A	SRX821383
**MDH_2014_ 00656**	Porcine	USA:MN	North America	2005	II	A	A1	A	SRX878521
**MDH_2014_ 00696**	Porcine	USA:MN	North America	2006	II	A	A1	A	SRR2082815
**MDH_2014_ 0153**	Porcine	USA:MN	North America	2006	II	A	A1	A	SRX517539
**MDH_2014_ 0169**	Porcine	USA:MN	North America	2007	II	A	A1	A	SRX515714
**MDH_2014_ 0180**	Porcine	USA:MN	North America	2007	III	B	B1	B	SRX495380
**NY_22699**	Porcine	USA:MD	North America	2002	II	A	A1	A	SRX652291
**NY_29768**	Porcine	USA:OR	North America	2003	II	A	A1	A	SRX707388
**NY_swgs11 08**	Porcine	USA:MN	North America	2008	II	A	A1	A	SRX476521
**NY_swgs11 66**	Porcine	USA:MN	North America	2010	II	A	A1	A	SRX529490
**NY_swgs11 77**	Porcine	USA:MN	North America	2011	II	A	A1	A	SRX547074
**NY_swgs11 80**	Porcine	USA:MN	North America	2011	III	B	B2	B	SRX569842
**NY_swgs11**	Porcine	USA:MN	North	2012	II	A	A1	A	SRX569843

**87**			America						
**NY_swgs1193**	Porcine	USA:MN	North America	2012	II	A	A1	A	SRX529479
**POR1_0012**	Porcine	USA:TX	North America	2013	III	B	B2	B	SRX712967
**POR1_0013**	Porcine	USA:TX	North America	2013	III	B	B2	B	SRX732849
**POR1_0019**	Porcine	USA:TX	North America	2014	II	A	A1	A	SRX743618
**POR1_0028**	Porcine	USA:TX	North America	2014	II	A	A1	A	SRX748929
**VA_WGS_00332**	Porcine	USA:VA	North America	2011	III	B	B1	B	SRX738212
**WAPHL_SAL_A00330**	Porcine	USA:MD	North America	2002	II	A	A1	A	SRX512394
**0207-107**	Porcine	USA:PA	North America	2002	?**	A	A2	A	Yue et al., 2012
**AUCVM_3596**	Bovine	USA:FL	North America	2005	II	A	A1	A	SRX838812
**AZ_TG68372**	Bovine	USA:UT	North America		II	A	A1	A	SRX378367
**AZ_TG68376**	Bovine	USA:UT	North America		II	A	A1	A	SRX378370
**AZ_TG68380**	Bovine	USA:UT	North America		II	A	A1	A	SRX378327
**AZ_TG68384**	Bovine	USA:UT	North America		II	A	A1	A	SRX378361
**CFSAN023385**	Bovine	USA:MN	North America	2013	II	A	A1	A	SRX696110

**CFSAN023386**	Bovine	USA:MN	North America	2013	II	A	A1	A	SRX696066
**CFSAN023387**	Bovine	USA:MN	North America	2013	II	A	A1	A	SRX696169
**CVM19593**	Bovine	Mexico	North America		II	A	A1	A	AHUD00000000
**IEH_NGS_SAL_00516**	Bovine	USA:WA	North America	2007	II	A	A1	A	SRX581722
**IEH_NGS_SAL_00560**	Bovine	USA:UT	North America	2007	II	A	A1	A	SRX672482
**IEH_NGS_SAL_00625**	Bovine	USA:ID	North America	2007	II	B	A1	A	SRX672536
**IEH_NGS_SAL_00637**	Bovine	USA:WA	North America	2007	II	B	A1	A	SRX641516
**IEH_NGS_SAL_00664**	Bovine	USA:AL	North America	2007	II	A	A1	A	SRX641694
**IEH_NGS_SAL_00689**	Bovine	USA:WA	North America	2007	II	A	A1	A	SRX641647
**IEH_NGS_SAL_00760**	Bovine	USA:WA	North America	2007	II	A	A1	A	SRX649212
**IEH_NGS_SAL_00789**	Bovine	USA:WA	North America	2007	II	A	A1	A	SRX649208
**IEH_NGS_SAL_00805**	Bovine	USA:OR	North America	2007	II	A	A1	A	SRX652331
**IEH_NGS_SAL_00859**	Bovine	USA:WA	North America	2007	II	A	A1	A	SRX656681
**MDH_2013_00017**	Bovine	USA:MN	North America	2007	II	A	A1	A	SRX476670
**MDH_2013_**	Bovine	USA:MN	North	2012	II	A	A1	A	SRX390888

**00182**			America						
**MDH_2014_ 0123**	Bovine	USA:MN	North America	2005	II	A	A1	A	SRX528045
**MDH_2014_ 0127**	Bovine	USA:MN	North America	2005	II	A	A1	A	SRX523503
**MDH_2014_ 0132**	Bovine	USA:MN	North America	2006	II	A	A1	A	SRX523522
**MDH_2014_ 0140**	Bovine	USA:MN	North America	2006	II	A	A1	A	SRX520655
**MDH_2014_ 0156**	Bovine	USA:MN	North America	2006	II	A	A1	A	SRX517517
**MDH_2014_ 0159**	Bovine	USA:MN	North America	2006	II	A	A1	A	SRX517544
**MDH_2014_ 0165**	Bovine	USA:MN	North America	2007	II	A	A1	A	SRX515694
**NY_22707_ 28**	Bovine	USA:MD	North America	2002	II	A	A1	A	SRX652288
**NY_29461**	Bovine	USA:CA	North America	2003	II	A	A1	A	SRX661538
**NY_FSL_R6_ 0956**	Bovine	USA:NY	North America	2007	II	A	A1	A	SRX512438
**NY_FSL_R8_ 2900**	Bovine	USA:NY	North America	2008	II	A	A1	A	SRX517509
**NY_swgs11 09**	Bovine	USA:MN	North America	2008	II	A	A1	A	SRX476472
**NY_swgs11 16**	Bovine	USA:MN	North America	2008	II	A	A1	A	SRX529489
**NY_swgs11 33**	Bovine	USA:MN	North America	2008	II	A	A1	A	SRX529486

**NY_swgs1179**	Bovine	USA:MN	North America	2011	II	A	A1	A	SRX547104
**VA_WGS_00045**	Bovine	USA:VA	North America	2005	II	A	A1	A	SRX467147
**VA_WGS_00097**	Bovine	USA:VA	North America	2005	II	A	A1	A	SRX476390
**VA_WGS_00098**	Bovine	USA:VA	North America	2006	II	A	A1	A	SRX476466
**VA_WGS_00148**	Bovine	USA:VA	North America	2006	II	A	A1	A	SRX506541
**WAPHL_SAL_A00029**	Bovine	USA:WA	North America	2003	II	A	A1	A	SRX333971
**WAPHL_SAL_A00045**	Bovine	USA:WA	North America	2004	II	A	A1	A	SRX334030
**WAPHL_SAL_A00046**	Bovine	USA:WA	North America	2004	II	A	A1	A	SRX334031
**WAPHL_SAL_A00050**	Bovine	USA:WA	North America	2005	II	A	A1	A	SRX349426
**WAPHL_SAL_A00065**	Bovine	USA:WA	North America	2006	II	A	A1	A	SRX424968
**WAPHL_SAL_A00067**	Bovine	USA:WA	North America	2006	II	A	A1	A	SRX424946
**WAPHL_SAL_A00071**	Bovine	USA:WA	North America	2006	II	A	A1	A	SRS528662
**WAPHL_SAL_A00212**	Bovine	USA:WA	North America	2006	II	A	A1	A	SRS562431
**WAPHL_SAL_A00302**	Bovine	USA:WA	North America	2002	II	A	A1	A	SRX497526
**WAPHL_SAL**	Bovine	USA:WA	North	2002	II	A	A1	A	SRX499447

**_A00317**			America						
**CFSAN023387**	Bovine	USA:MN	North America	2013	II	A	A1	A	SRX696169
**FCC0034**	Bovine	Nicaragua	Southe America	2010	III	B	B1	B	SRX385860
**USMARC-S3124**	Bovine	USA	North America	2011	II	A	A1	A	CP006631.1
**CVM22513**	Bovine	USA:NC	North America	2003	II	A	A1	A	AHTU00000000
**USMARC-1927**	Bovine	USA	North America	2011	III	B	B2	B	CP007216.1
**0007-33**	Bovine	USA:PA	North America	2000	?**	A	A1	A	Yue et al., 2012
**0007-407**	Bovine	USA:PA	North America	2000	?**	A	B1	A	Yue et al., 2012
**0008-169**	Bovine	USA:PA	North America	2000	?**	B	B1	B	Yue et al., 2012
**0008-254**	Bovine	USA:PA	North America	2000	?**	A	A1	A	Yue et al., 2012
**0008-303**	Bovine	USA:PA	North America	2000	?**	A	A2	A	Yue et al., 2012
**0008-322**	Bovine	USA:PA	North America	2000	?**	A	A1	A	Yue et al., 2012
**0101-406**	Bovine	USA:PA	North America	2001	?**	A	A1	A	Yue et al., 2012
**0101-59**	Bovine	USA:PA	North America	2001	?**	B	B2	B	Yue et al., 2012
**0102-163**	Bovine	USA:PA	North America	2001	?**	A	A1	A	Yue et al., 2012

**0107-158**	Bovine	USA:PA	North America	2001	?**	A	A2	A	Yue et al., 2012
**0108-436**	Bovine	USA:PA	North America	2001	?**	A	A2	A	Yue et al., 2012
**0207-322**	Bovine	USA:PA	North America	2002	?**	A	A1	A	Yue et al., 2012
**0211-109**	Bovine	USA:PA	North America	2002	?**	A	A2	A	Yue et al., 2012
**0306-88**	Bovine	USA:PA	North America	2003	?**	A	A1	A	Yue et al., 2012
**0205-86**	Bovine	USA:PA	North America	2002	?**	B	B1	B	Yue et al., 2012

* Indicates the lineages (I, II or III) of the S. Newport strains (Sangal V, Harbottle H, Mazzoni CJ, Helmuth R, Guerra B, Didelot X, Paglietti B, Rabsch W, Brisse S, Weill FX, Roumagnac P, Achtman M. 2010. Evolution and population structure of Salmonella enterica serovar Newport. J Bacteriol 192:6465-76).

** Strain of undetermined lineage (Yue M, Schmieder R, Edwards RA, Rankin SC, Schifferli DM. 2012. Microfluidic PCR Combined with Pyrosequencing for Identification of Allelic Variants with Phenotypic Associations among Targeted Salmonella Genes. Appl Environ Microbiol 78:7480-2).
