# Supplementary figures and images for: Cooperation of Adhesin Alleles in Salmonella-Host Tropism
Source: mSphere. 2017 Mar 8;2(2):e00066-17. doi: 10.1128/mSphere.00066-17 (PMC5343171; doi:10.1128/mSphere.00066-17)

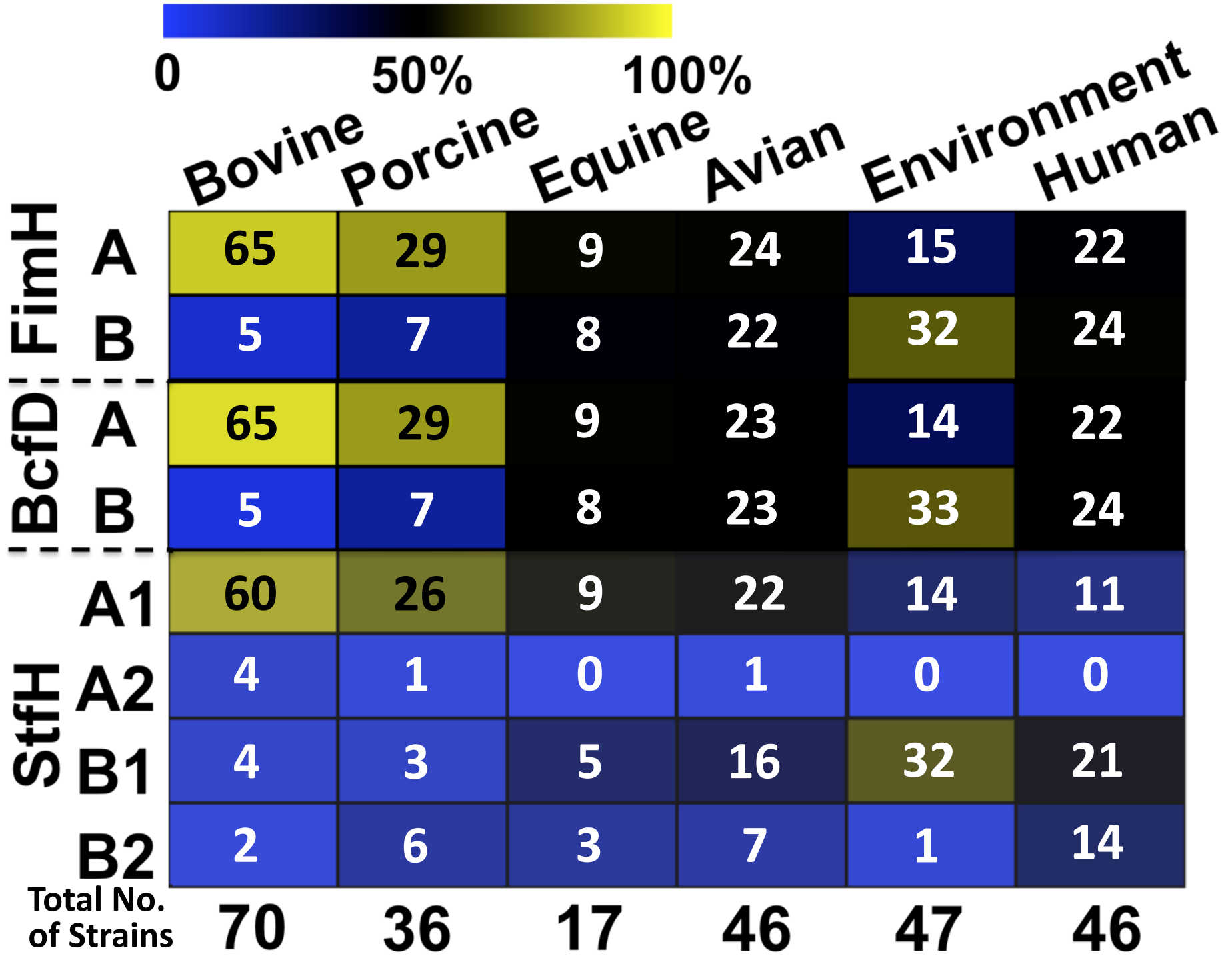

Supplement: FIG S1 [file sph002172250sf6.pdf]

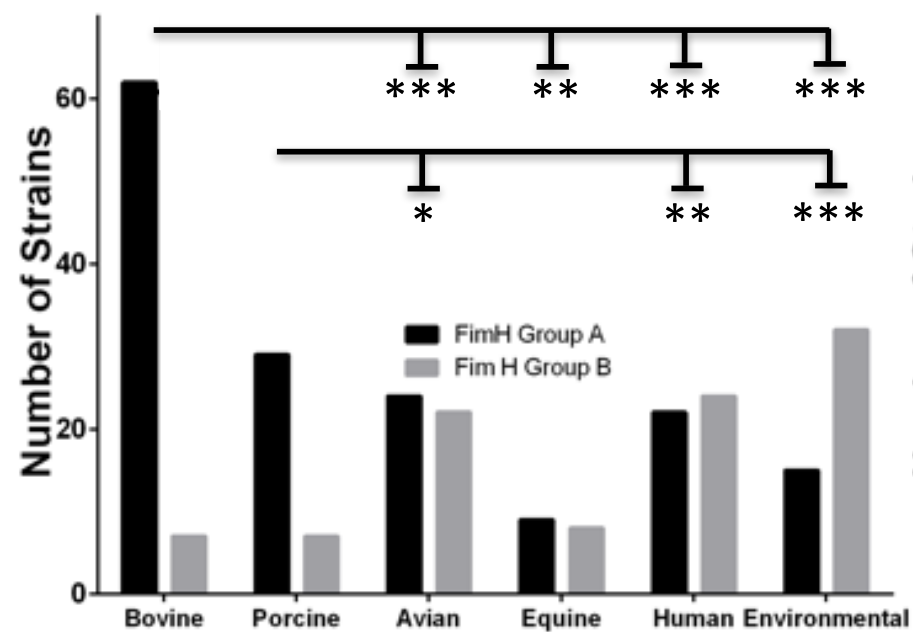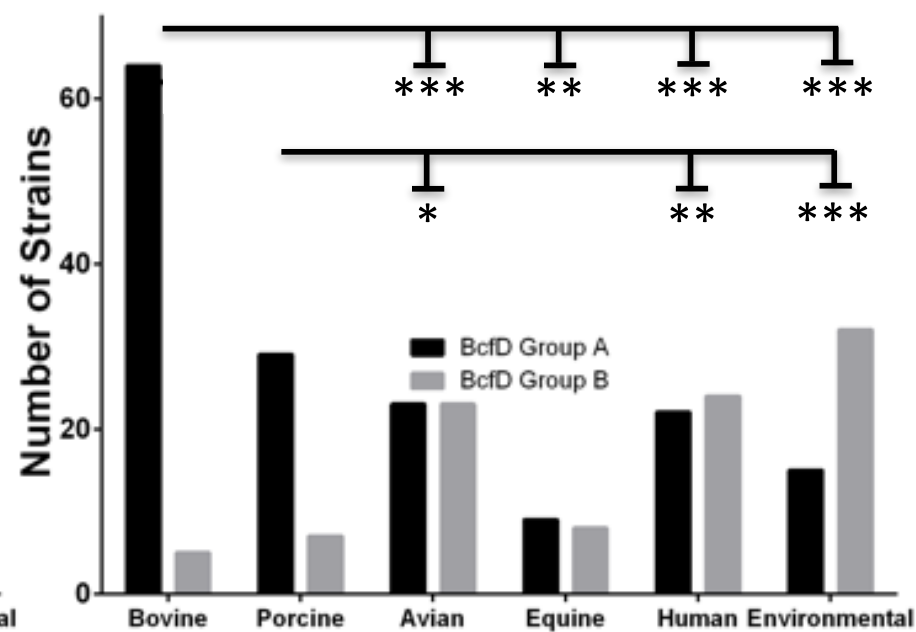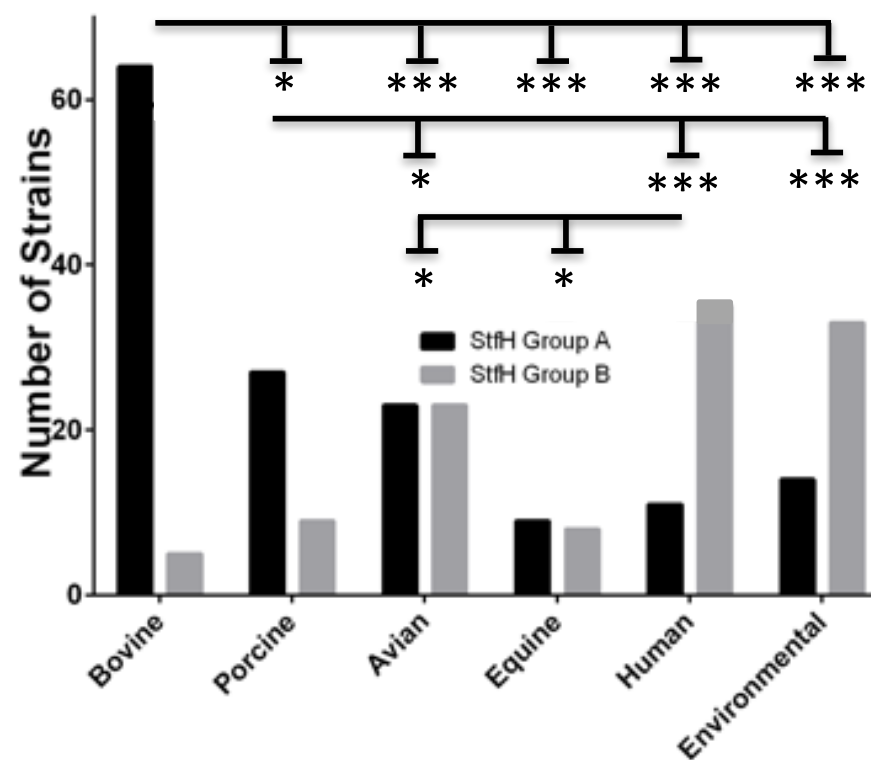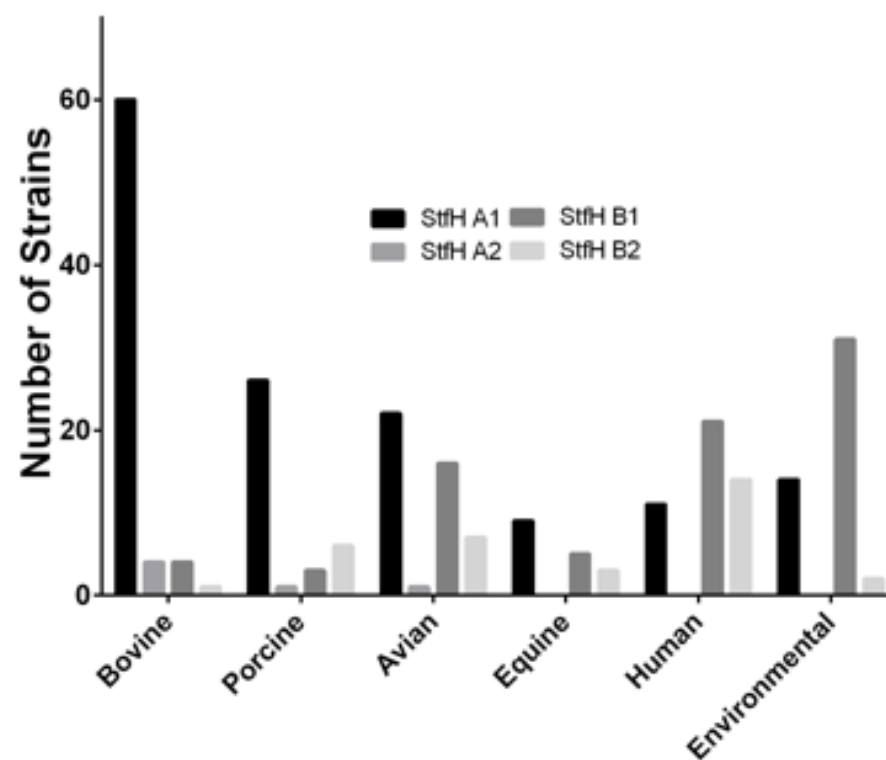

Supplement: FIG S2 [file sph002172250sf7.pdf]

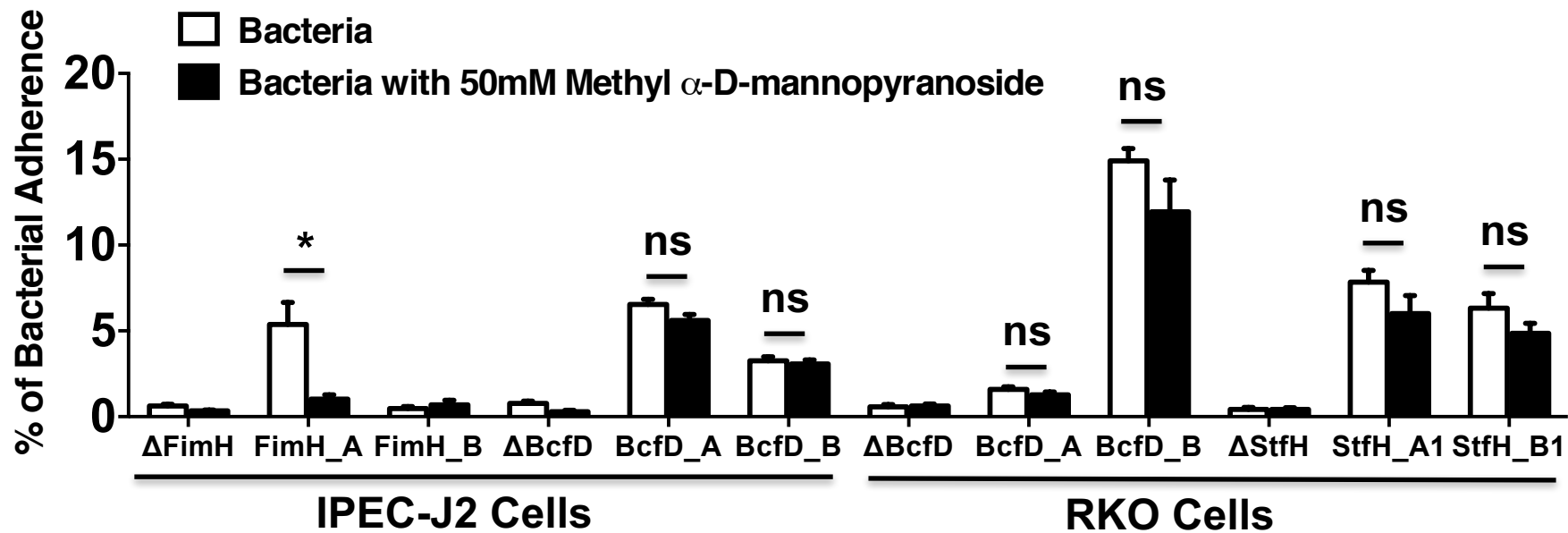

Supplement: FIG S3 [file sph002172250sf8.pdf]
