# Supplementary material for: Cooperation of Adhesin Alleles in Salmonella-Host Tropism
Source: mSphere. 2017 Mar 8;2(2):e00066-17. doi: 10.1128/mSphere.00066-17 (PMC5343171; doi:10.1128/mSphere.00066-17)
Supplement: TABLE S2 [file sph002172250st2.pdf]

Table S2A: Statistical data (P-values) of 2x2 contingency tables (Fisher's Exact Test) with strain numbers separated by sources and FimH alleles.

FimH Host Group vs (at right)	Porcine	Avian	Equine	Human	Environmental
Bovine	0.2294	**7.61E-06**	**0.001348**	**1.02E-06**	**7.73E-11**
Porcine	X	**0.01035**	0.05256	**0.002933**	**1.18E-05**
Avian	X	X	1	0.835	0.05983
Equine	X	X	X	0.7816	0.1511
Human	X	X	X	X	0.1408
Environmental	X	X	X	X	X

Tests were performed with the number of Group A and B isolates for the group at left vs the group listed at the top. Values with $P<0.05$ are **underlined and in bold**.

Table S2B: Statistical data (P-values) of 2x2 contingency tables (Fisher's Exact Test) with strain numbers separated by sources and BcfD alleles.

BcfD Host Group vs (at right)	Porcine	Avian	Equine	Human	Environmental
Bovine	0.1025	**2.56E-07**	**0.00339**	**8.69E-08**	**3.18E-12**
Porcine	X	**0.005587**	0.0526	**0.002933**	**1.18E-05**
Avian	X	X	1	1	0.09342
Equine	X	X	X	0.7816	0.1511
Human	X	X	X	X	0.1408
Environmental	X	X	X	X	X

Tests were performed with the number of Group A and B isolates for the group at left vs the group listed at the top. Values with $P<0.05$ are **underlined and in bold**.

Table S2C: Statistical data (P-values) of 2x2 contingency tables (Fisher's Exact Test) with strain numbers separated by sources and StfH alleles.

StfH Host group vs (at right)	Porcine	Avian	Equine	Human	Environmental
Bovine	**0.01599**	**2.56E-07**	**0.000339**	**1.06E-14**	**6.92E-13**
Porcine	X	**0.01305**	0.127	**5.55E-06**	**5.78E-05**
Avian	X	X	1	**0.01689**	0.0579
Equine	X	X	X	**0.03671**	0.1392
Human	X	X	X	X	0.6411
Environmental	X	X	X	X	X

Tests were performed with the number of Group A and B isolates for the group at left vs the group listed at the top. Values with $P<0.05$ are **underlined and in bold**.
