# Supplementary material for: Cooperation of Adhesin Alleles in Salmonella-Host Tropism
Source: mSphere. 2017 Mar 8;2(2):e00066-17. doi: 10.1128/mSphere.00066-17 (PMC5343171; doi:10.1128/mSphere.00066-17)
Supplement: TABLE S3 [file sph002172250st3.pdf]

Table S3A: Statistical data (unpaired t-tests, * P < 0.05, ** P < 00.1, *** P < 0.001) for the numbers of adherent strains with different FimH alleles that bind to each intestinal epithelial cell line (Ø, nonfimbriated empty vector control strain).

FimH	IPEC-J2		CMS		J8		RKO		Caco-2	
Alleles	Ø	A	Ø	A	Ø	A	Ø	A	Ø	A
A	***		***		***		***		*	
B	*	***	***	***	**	***	***	***	***	***

Table S3B: Statistical data (unpaired t-tests, * P < 0.05, ** P < 00.1, *** P < 0.001) for the numbers of adherent strains with different BcfD alleles that bind to each intestinal epithelial cell line (Ø, nonfimbriated empty vector control strain).

BcfD	IPEC-J2		CMS		J8		RKO		Caco-2	
Alleles	Ø	A	Ø	A	Ø	A	Ø	A	Ø	A
A	***		-		***		**		-	
B	**	*	-	-	**	-	***	***	-	-

Table S3C: Statistical data (unpaired t-tests, * P < 0.05, ** P < 00.1, *** P < 0.001) for the numbers of adherent strains with different StfH alleles that bind to each intestinal epithelial cell line (Ø, nonfimbriated empty vector control strain).

StfH	IPEC-J2				CMS				J8				RKO				Caco-2			
Alleles	Ø	A1	A2	B1	Ø	A1	A2	B1	Ø	A1	A2	B1	Ø	A1	A2	B1	Ø	A1	A2	B1
A1	***				-				***				***				-			
A2	***	-			***	***			-	**			-	***			-	-		
B1	**	**	***		**	*	-		-	**	-		-	*	-		-	-	-	
B2	***	-	-	**	*	-	-	-	-	**	-	*	***	-	*	-	-	-	-	-
