# Supplementary material for: Cooperation of Adhesin Alleles in Salmonella-Host Tropism
Source: mSphere. 2017 Mar 8;2(2):e00066-17. doi: 10.1128/mSphere.00066-17 (PMC5343171; doi:10.1128/mSphere.00066-17)
Supplement: TABLE S4 [file sph002172250st4.pdf]

Table S4. List of Strains and Plasmids used in this study.

Strain	Genotype	Source
AAEC189	*E. coli*	(1)
SE5000	*E. coli*	(2)
AJB3	*S. Typhimurium* SR11 Nalr	(3)
AJB4	AJB3 *fim*	(3)
STN35	*S. Typhimurium bcfC*::miniTn5, Kmr	(4)
AJB4 *bcfC*::mTn5	AJB4 *bcfC*::miniTn5, Kmr	This study
SL254	*S.* Newport human isolate with Group A *bcfD* and *fimH* alleles and Group A1 *stfH* allele	(5)
SL317	*S.* Newport human isolate with Group B *bcfD* and *fimH* alleles and Group B1 *stfH* allele	(5)
0109-1163	*S.* Newport environmental isolate with Group B *bcfD* and *fimH* alleles and Group B2 *stfH* allele	(5)
0209-48	*S.* Newport feline isolate with Group A *bcfD* and *fimH* alleles and Group A2 *stfH* allele	(5)
**Plasmid**	**Description**	**Source**
pMAL-c2x	Expression plasmid	New England Biolabs, Inc.
pACYC184	ColE1 compatible cloning vector	(6)
pAZ30	*fimH* from *S.* Newport strain 41 cloned into *Bam*HI and *Nde*I sites of pMAL-c2x	(7)
pAZ30-fim45	*fimH* from *S.* Newport strain 45 cloned into *Bam*HI and *Nde*I sites of pMAL-c2x	(7)
pAZ37	*fimA* through *fimF*, Δ*fimH*, cloned into *Bam*HI site of pACYC184	(8)
pBAD33	Arabinose inducible cloning vector	(9)
pHSG576	A very low copy number vector	(10)
pLDHSG-Bcf-S	pHSG-576 with *bcfA*-G from *S.* Newport strain SL254 cloned into the *Bam*HI/*Hin*dIII sites	This study
pLDHSG-Bcf-L	pHSG-576 with two DNA binding protein immediately upstream and downstream of the *bcf* gene cluster and *bcfA*-H from *S.* Newport strain SL254 cloned into the *Bam*HI/*Hin*dIII sites	This study
pLDHSG-Bcf-B	pLDHSG-Bcf-S with the *bcfD* gene replaced by the *bcfD* gene from SL317 by Gibson cloning	This study
pLDBAD-Stf	pBAD33 with complete *stf* gene cluster from *S.* Newport strain SL254 cloned into multiple cloning site (MCS)	This study
pLDBAD-StfH-SL317	pLDBAD-Stf with *stfH* gene replaced with *stfH* allele from *S.* Newport strain SL317	This study
pLDBAD-StfH-	pLDBAD-Stf with *stfH* gene replaced with *stfH*	This study

0109	allele from *S.* Newport strain 0109-1163	
pLDBAD-StfH-0209	pLDBAD-Stf with *stfH* gene replaced with *stfH* allele from *S.* Newport strain 0209-48	This study

1. Blomfield IC, McClain MS, Eisenstein BI. 1991. Type 1 fimbriae mutants of *Escherichia coli* K12: characterization of recognized afimbriate strains and construction of new *fim* deletion mutants. Mol Microbiol 5:1439-1445.
2. Silhavy TJ, Berman ML, Enquist LW. 1984. Experiments with gene fusion. Cold Spring Harbor Laboratory, Cold Spring Harbor, N.Y.
3. Baumler AJ, Tsolis RM, Bowe FA, Kusters JG, Hoffmann S, Heffron F. 1996. The pef fimbrial operon of *Salmonella typhimurium* mediates adhesion to murine small intestine and is necessary for fluid accumulation in the infant mouse. Infect Immun 64:61-8.
4. Tsolis RM, Townsend SM, Miao EA, Miller SI, Ficht TA, Adams LG, Baumler AJ. 1999. Identification of a putative *Salmonella enterica* serotype typhimurium host range factor with homology to IpaH and YopM by signature-tagged mutagenesis. Infect Immun 67:6385-93.
5. Yue M, Schmieder R, Edwards RA, Rankin SC, Schifferli DM. 2012. Microfluidic PCR Combined with Pyrosequencing for Identification of Allelic Variants with Phenotypic Associations among Targeted *Salmonella* Genes. Appl Environ Microbiol 78:7480-2.
6. Chang AC, Cohen SN. 1978. Construction and characterization of amplifiable multicopy DNA cloning vehicles derived from the P15A cryptic miniplasmid. J Bacteriol 134:1141-56.
7. Yue M, Han X, Masi LD, Zhu C, Ma X, Zhang J, Wu R, Schmieder R, Kaushik RS, Fraser GP, Zhao S, McDermott PF, Weill FX, Mainil JG, Arze C, Fricke WF, Edwards RA, Brisson D, Zhang NR, Rankin SC, Schifferli DM. 2015. Allelic variation contributes to bacterial host specificity. Nat Commun 6:8754.
8. Guo A, Lasaro MA, Sirard J-C, Kraehenbühl J-P, Schifferli DM. 2007. Adhesin-dependent binding and uptake of *Salmonella enterica* serovar Typhimurium by dendritic cells. Microbiology 153:1059-1069.
9. Guzman LM, Belin D, Carson MJ, Beckwith J. 1995. Tight regulation, modulation, and high-level expression by vectors containing the arabinose PBAD promoter. J Bacteriol 177:4121-4130.
10. Takeshita S, Sato M, Toba M, Masahashi W, Hashimoto-Gotoh T. 1987. High-copy-number and low-copy number plasmid vectors for *lacZ* alpha-complementation and chloramphenicol- or kanamycin-resistance selection. Gene 61:63-74.
