# Supplementary material for: Cooperation of Adhesin Alleles in Salmonella-Host Tropism
Source: mSphere. 2017 Mar 8;2(2):e00066-17. doi: 10.1128/mSphere.00066-17 (PMC5343171; doi:10.1128/mSphere.00066-17)
Supplement: TABLE S5 [file sph002172250st5.pdf]

Table S5: Primers used for PCR in this study.

PCR Primers	Sequence	Function
StfWholeNew FWD	GGTGGTTCTAGACGTAAAAATACTCACCAACTTTTAATAA	Cloning of Stf cluster into pBAD33
StfWholeNew REV	GGTGGTAAGCTTTCATAAATACTCAAATGTAACTAGTGC	Cloning of Stf cluster into pBAD33
StfHGibsonNewFWD	CTGATTCCGGGAACGTTCTTCGCAAGCGCACTAGTACTAGT TACATTTGAGTATTTATGAATG	Replacement of *stfH* allele in pLDBAD-Stf
StfHGibsonNewREV	CTGAAAATCTTCTCTCATCCGCCAAAACAGCCAAGCTTAAG CTTTCTAGACACTAAATCTC	Replacement of *stfH* allele in pLDBAD-Stf
BcfpiecepBR322FWD	GGTGGTAAGCTTCCCTTATTTTTATATTTAAAAGGAGC	Cloning of Bcf gene cluster from *bcfA* through *bcfG* into pHSG-576; primer was originally designed for cloning into pBR322
BcfGREV	GGTGGTGGATCCTTAATGAATACGCGTCAGATCC	Cloning of Bcf gene cluster from *bcfA* through *bcfG* into pHSG-576
BcfDNAFWD	GGTGGTAAGCTTCATTGAGTAGACAACCGTTA	Cloning of Bcf gene cluster with DNA binding protein at 5' end through *bcfH* into pHSG-576
BcfpHSG576 REVNew	AATAATGGATCCTCACCCCTTCGCTTTCT	Cloning of Bcf gene cluster with DNA binding protein at 5' end through *bcfH* into pHSG-576
GibpBADBcfE FWD	CTTTGACTAAATGAACAGATCACACTGCG	Inverse PCR of pLDHSG-Bcf-S with *bcfD* deletion
GibpBADBcfA REV	CGATAAGGAATCAGGAATAAACCATGCTAAATG	Inverse PCR of pLDHSG-Bcf-S with *bcfD* deletion
GibpBADBcfD FWD	GTGTCATTAAATGAAAATACCTCTTTTATTTGC	Replacement of *bcfD* allele in pLDHSG-Bcf-S
GibpBADBcfD REV	ATCTGTTCATTTAGTCAAAGTCCACTCGC	Replacement of *bcfD* allele in pLDHSG-Bcf-S
